# Information-Theoretic Measures of Metacognition: Bounds and Relation to Group Performance

**DOI:** 10.1162/OPMI.a.40

**Published:** 2025-10-17

**Authors:** Sascha Meyen, Frieder Göppert, Carina Schrenk, Ulrike von Luxburg, Volker H. Franz

**Affiliations:** Department of Computer Science, University of Tübingen, Tübingen, Germany; Tübingen AI Center, Tübingen, Germany

**Keywords:** metacognition, metainformation, signal detection theory, confidence weighted majority voting, group decisions

## Abstract

Metacognition comprises the ability to differentiate the accuracy of predictions about the world. This is often called Type 2 performance (with Type 1 performance being the overall accuracy). Typical measures of metacognition are based on signal detection theory and require the strong assumption of truncated normal noise underlying confidence ratings. To minimize distributional assumptions, measures based on classical information theory have been proposed. We further this approach by providing bounds on its key quantity, the transmitted information. We show that classifiers making predictions with a certain accuracy can transmit information only within a limited range, depending on the underlying noise distribution: The lowest transmitted information indicates the worst Type 2 performance and corresponds to binary noise; the highest transmitted information indicates the best Type 2 performance and corresponds to uniform noise. Because normal noise is only an intermediate case, traditional measures based on this assumption can bias interpretations of Type 2 performance. Based on these bounds, we suggest a new measure: Relative metainformation (RMI). RMI scales from 0 (lower bound) to 1 (upper bound) and therefore advances towards the much-needed decoupling of Type 2 from Type 1 performance measures. To demonstrate the strengths of RMI, we apply it to groups: In a setting where multiple independent group members with fixed accuracies combine their predictions in an optimal way, we show that the group performance depends directly on RMI: Group accuracy is best vs. worst if the group members have highest vs. lowest RMI values. Overall, our theoretical bounds allow to better evaluate measures of Type 2 and group performance.

## INTRODUCTION

Humans have the ability to differentiate levels of uncertainty in their mental states. This ability to form cognition about other cognitive states—metacognition—underpins how we orient ourselves in the world (Ptasczynski et al., 2022; Schulz et al., 2023): It determines, for example, which questions we ask in academic discourse and which sections of a scientific paper we reread for better understanding.

Metacognition research has recently gained traction. Currently, one of the goals is to find appropriate measures of metacognitive abilities (Boundy-Singer et al., 2023; Guggenmos, 2022; Katyal & Fleming, 2024; Rahnev, 2025; Shekhar & Rahnev, 2024). In general, metacognition research differentiates between two measurable quantities: (1) the ability to predict states in the world correctly vs. (2) the second-order metacognitive ability to assess one’s own accuracy. These two quantities are called Type 1 vs. Type 2 performance, respectively (Clarke et al., 1959; Fleming & Lau, 2014; Maniscalco & Lau, 2014). Independent of how accurate predictions are, one human rater may know which of their predictions are highly certain and which are prone to be erroneous (high Type 2 performance) while another rater may fail in making this distinction between more vs. less certain predictions (low Type 2 performance).

As of now, the most prominent measures of Type 2 performance are meta−*d*′ and *M*−ratio (Fleming & Lau, 2014; Maniscalco & Lau, 2012, 2014). They are based on signal detection theory (Green & Swets, 1988) and – similar to their Type 1 performance counterpart sensitivity *d*′– assume underlying normal noise for Type 1 responses (i.e., predictions). This assumption is plausible due to the central limit theorem suggesting that the average activation of neuron populations converges to normal distributions (Softky & Koch, 1993; Usher & McClelland, 2001). However, Rausch et al. (2023) showed that the noise model of Type 2 responses (i.e., confidence ratings) assumed by meta−*d*′ follows an independent truncated normal distribution. This, in turn, is not supported by the central limit theorem argument. Furthermore, Shekhar and Rahnev (2021, 2024) presented empirical data suggesting deviations from normally distributed noise underlying confidence ratings.

The reliance on distributional assumptions creates problems when using these measures to compare different tasks and classifiers. While the normal noise assumption seems plausible for the frequently used dot motion tasks creating a gradual sense of confidence (e.g., Rouault et al., 2019), general knowledge question tasks can produce bimodal confidence distributions reflecting that participants either do or do not know the answer (see e.g., Little, 2023). The latter case deviates from the (truncated) normal noise assumptions and such model-misspecifications can bias results. In the same way, comparisons of Type 2 performance between humans and algorithmic classifiers (e.g., in deep neural networks for vision, Bowers et al., 2022) are problematic when underlying noise distributions differ. Such comparisons are inherently limited when the measures are based on models tailored towards one of the two comparands.

These limitations may be overcome by Dayan (2023)’s recently suggested information-theoretic measures of Type 2 performance. Information-theoretic measures quantify Type 2 performance (without distributional assumptions) as the amount of information that confidence ratings transmit about the accuracy of individual predictions. Here, we develop this information-theoretic approach further. Our contribution is to provide tight upper and lower bounds for the amount of transmitted information. These bounds allow us to appropriately normalize the information-theoretic Type 2 measures making interpretations easier. With this, we introduce a new measure, the relative metainformation RMI, which assigns values around 1 to the best possible Type 2 performance for a given Type 1 performance and values around 0 to the worst. This approach contributes to removing the problematic confound between measures of Type 1 and Type 2 performance that befalls existing measures.

Type 2 performance is an important aspect when it comes to making decisions in groups (Bahrami et al., 2010; Fleming, 2024; Frith, 2012; Frith & Frith, 2012, on pp. 247–249; Meyen et al., 2021): When individual group members can differentiate between high vs. low certainty in their predictions (high Type 2 performance), the group can weigh individual predictions accordingly to make better group decisions. Extending prior theoretical work on confidence weighted majority voting (CWMV, Grofman et al., 1983; Nitzan & Paroush, 1982), we provide bounds on the group performance in a setting in which each individual prediction comes with its particular confidence. Interestingly, these group bounds are related to the information bounds: Individual group members with the highest (RMI = 1) vs. lowest Type 2 performance (RMI = 0) also contribute maximally vs. minimally to the Type 1 group performance, that is, how often the group makes a correct decision. We thereby introduce a formal tie between group decisions and metacognition.

We proceed by introducing our mathematical setting, giving an illustrative example for it, and providing bounds that the accuracy (a Type 1 performance measure) imposes on the transmitted information (a key quantity in Type 2 performance measures). With these bounds, we then propose the new information-theoretic Type 2 measure, RMI. Finally, we demonstrate its tie to the Type 1 performance in groups.

## SETTING AND NOTATION

We consider a setting in which a classifier, be it a human rater performing a discrimination task or an algorithmic classifier in a classification application (e.g., a deep neural network classifier), predicts a true label *Y*. We use uppercase letters to denote random variables (*Y*) and lowercase letters for their realizations (*y*). We begin by assuming that there are only two labels (two options when human raters decide in a metacognition experiment, e.g., whether more dots are presented on the left or on the right side of the screen as in Rouault et al., 2019). Later, we will generalize this to a finite number of labels, *L*.

**Assumption 1** (Binary Label). *y* ∈ {−1, +1}

We assume the prior probabilities for these labels to be known. In a typical metacognition experiment, this assumption is guaranteed by the experimenter fixing how often each stimulus category is presented. For simplicity, we assume the *y* = +1 label to have the higher (or equal) prior probability and denote it by *p*_+_. In the simplest case, both labels have the same prior probability *P*(*Y* = +1) = *P*(*Y* = −1) with *p*_+_ = 0.5.

**Assumption 2** (Known Prior). *p*_+_ = *P*(*Y* = +1), *p*_+_ ≥ 0.5

A classifier outputs a prediction $\hat{Y}$ and a confidence rating *C*, which we jointly refer to as the classifier’s response, $\left(\hat{Y},C\right)$. For our theoretical derivations, we assume these confidence ratings to be calibrated. That is, they reflect the posterior probability of the prediction being correct. In other words, we assume the conditional accuracy of the classifier’s particular prediction to be known. This has been dubbed the *probability elicitation* setting (Masnadi-Shirazi, 2013; Savage, 1971) and clearly is a very strong assumption (which is nevertheless regularly made; Baan et al., 2022; Kull et al., 2019; Lin et al., 2022). In practice, human and algorithmic classifiers can only estimate these posterior probabilities. Therefore, when we later define a measure of Type 2 performance, we approximate this assumption by replacing given responses by estimates of the posterior probability: For each response (e.g., “prediction $\hat{Y}=+1$ with high confidence”), we estimate the accuracy from the relative frequency of correct responses. This is in line with the approach of Dayan (2023). Note that this replaces a classifier’s actual confidence responses and, as a consequence, information-theoretic measures of Type 2 performance only measure the ability of a classifier to differentiate between levels of uncertainty. They do not evaluate how well participants can numerically estimate the probability of being correct. This has consequences for the interpretation of information-theoretic measures of Type 2 performance, which we will discuss later. For now, the perfect calibration assumption is a tool to derive bounds of these information-theoretic measures.

**Assumption 3** (Perfect Calibration). $c=P\left(Y=\hat{y}\;|\;\hat{Y}=\hat{y},C=c\right)$

Finally, we assume predictions to be Bayes optimal. That is, classifiers will predict the label with the highest posterior probability: Rather than outputting prediction $\hat{Y}=+1$ with a confidence rating of *C* = 40%, a classifier would predict the opposite label $\hat{Y}=-1$ with the higher confidence rating *C* = 60%. At first, it may even seem counter-intuitive that a classifier would ever make a prediction with a confidence rating below 50% but there are experimental designs in which human raters first give a fast prediction and then, after gathering postdecisional evidence, rate their confidence (e.g., Navajas et al., 2016). In these cases, human raters sometimes report confidence below 50% because they realize that their initial prediction was likely incorrect. We assume here that the prediction is then simply re-coded. This is only a mild additional assumption given that we already assume the classifier to know the posterior probability of their prediction being correct due to perfect calibration. It entails that confidence ratings are always at least 50%.

**Assumption 4** (Bayes Predictions). *c* ≥ 0.5

### Confidence Distributions Determine Performance

We now turn to evaluating the performance of classifiers under these assumptions. We consider two performance measures: the accuracy and the transmitted information (often called the mutual information). We only make assumptions on the classifier’s responses, prediction $\hat{Y}$ and confidence rating *C*. We are not interested in the internal mechanisms of a classifier so that we can derive a model-free Type 2 performance measure later. Thus, the performance of a classifier in our setting is determined by its response distribution, $f_{\hat{Y},C}\left(\hat{y},c\right)$, which indicates for each prediction-confidence pair how often it is output by the classifier. Further, the performance of a classifier will be entirely determined by its (marginal) *confidence distribution*, $f_{C}\left(c\right)=f_{\hat{Y},C}\left(+1,c\right)+f_{\hat{Y},C}\left(-1,c\right)$, as we prove below. This confidence distribution indicates how often the classifier outputs a particular confidence rating *c*—regardless of its prediction. Note that our setting allows predictions for one label to come with (on average) higher confidence ratings than the other allowing cases in which one true label is easier to ascertain than the other.

### Accuracy

The accuracy acc is the probability of a classifier’s prediction to be correct, $acc=P\left(\hat{Y}=Y\right)$. Because we assume the confidence rating to be the probability *of a particular* prediction being correct, the overall accuracy is the expected value of the confidence rating *C*.

**Proposition 1**. *(Accuracy is the expected value of the confidence rating) Under* *Assumptions 1–4*,

$$acc=P\left(Y=\hat{Y}\right)=E\left[C\right].$$

**Figure 1.**
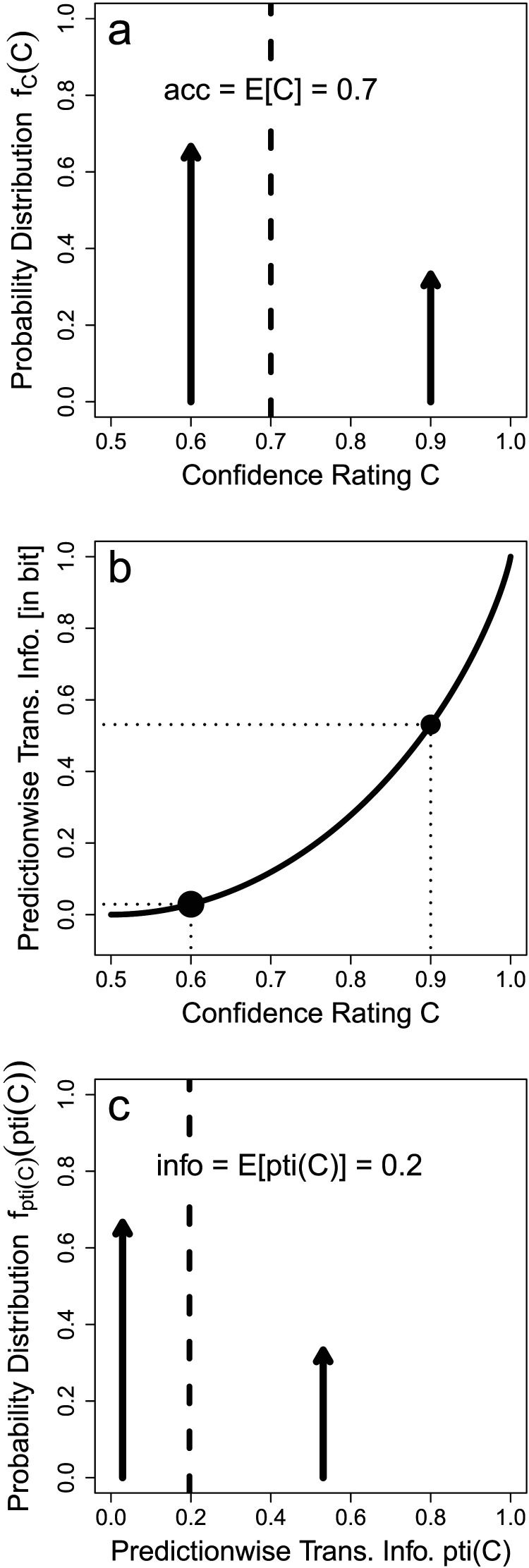
Relation Between Predictionwise Confidence Rating and Transmitted Information. *Note.* In our setting, each prediction comes with a confidence rating *C* indicating the accuracy (posterior probability) of that particular prediction being correct. Here, we show an example where a classifier produces predictions with low confidence rating (*c* = 60%) in 2/3 of the cases or high confidence rating (*c* = 90%) in 1/3 of the cases. Arrows indicate Dirac delta distributions with probability mass equal to their heights. (a) The expected value of these confidence ratings are the overall (or marginal) accuracy acc = *E*[*C*] as per Proposition 1. (b) Each predictionwise confidence rating *C* translates into a predictionwise transmitted information, pti(*C*), where we here assume for simplicity *p*_+_ = 0.5. (c) The expected value of these predictionwise transmitted information is then the overall (marginal) transmitted information info = *E*[pti(*C*)] as per Proposition 2.

See supplement for the proof. This situation is exemplified in Figure 1a where a classifier outputs predictions either with a low or a high confidence rating. Two-thirds of the predictions come with the low confidence rating of *c* = 60% and one-third come with the high confidence rating of *c* = 90%. Together, this entails an accuracy of $acc=E\left[C\right]=\frac{2}{3}\cdot 60\%+\frac{1}{3}\cdot 90\%=70\%$.

### Transmitted Information

Next to the accuracy, the performance of a classifier can also be measured by the information its responses transmit about the true labels. This is often also called the mutual information between response and true label (see Cover & Thomas, 2006, or MacKay, 2003, for a longer introduction to information theory). We will denote it by $\text{info}=I\left(Y;\hat{Y},C\right)$. While accuracy measures the probability of the prediction matching the true label, transmitted information measures how much uncertainty about the true label is in expectation reduced upon knowing the response of the classifier. Consider the baseline uncertainty, or entropy, about the true label, $H\left(Y\right)=\sum_{y}P\left(y\right)\log\frac{1}{P\left(y\right)}$, where we take the logarithm to the base 2 yielding the unit bit. Assuming binary labels, this can be written as

$$H\left(Y\right)=H_{2}\left(p_{+}\right)=p_{+}\log\left(\frac{1}{p_{+}}\right)+\left(1-p_{+}\right)\log\left(\frac{1}{1-p_{+}}\right).$$

Note that by convention the *p*_+_ = 100% case has an initial uncertainty of *H*_2_(1) = 0 bit because $\lim_{p_{+}\to 1}\left(1-p_{+}\right)\log\left(\frac{1}{1-p_{+}}\right)=0$.

Given a classifier’s response $\left(\hat{Y},C\right)$, this initial uncertainty reduces to the conditional entropy, $H\left(Y|\hat{Y},C\right)=E\left[\sum_{y}P\left(y|\hat{Y},C\right)\log\frac{1}{P\left(y|\hat{Y},C\right)}\right]$, where the expected value is over the possible responses $\left(\hat{Y},C\right)$. Again, with binary labels this simplifies to

$$H\left(Y|\hat{Y},C\right)=E\left[H_{2}\left(C\right)\right].$$

For example, a prior probability of *p*_+_ = 50% entails an initial uncertainty (entropy) of *H*_2_(*p*_+_) = *H*_2_(0.5) = 1 bit. If a classifier outputs the prediction $\hat{Y}=+1$ with confidence rating *C* = 70%, the remaining uncertainty is *H*_2_(70%) = 0.88 bit. Thus, the initial uncertainty about the identity of the true label is reduced upon receiving the classifier’s response by *H*_2_(0.5) − *H*_2_(0.7) = 1 − 0.88 = 0.12 bit. We denote this reduction of uncertainty for a particular response as the predictionwise transmitted information, pti(*C*) (often also called pointwise mutual information),

$$pti\left(C\right)=H_{2}\left(p_{+}\right)-H_{2}\left(C\right).$$

Quantifying uncertainty and predictionwise transmitted information in this way is natural in at least two senses. First, uncertainty is additive for independent random variables: While flipping a fair coin yields an uncertainty of 1 bit, flipping two fair coins yields an uncertainty of 2 bit. Second, as can be seen in Figure 1b, predictionwise transmitted information is a monotone and convex function of *C*. This entails that an increase in confidence from *C* = 50% to *C* = 55% corresponds to a lower gain in predictionwise transmitted information than an increase from *C* = 95% to *C* = 100%. Although in both cases, an additional 5% of the predictions are correct, reducing all remaining uncertainty in the latter case is—consistent with intuition—quantified as more information.

The information a classifier’s response transmits about the true label is the expected reduction in uncertainty over all possible responses, $I\left(Y;\hat{Y},C\right)=H\left(Y\right)-H\left(Y|\hat{Y},C\right)$. Thus, similar to the accuracy, it is entirely determined by the distribution over the confidence ratings *C*: The transmitted information is the expected value of the predictionwise transmitted information.

**Proposition 2**. *(Transmitted information is the expected value of the predictionwise transmitted information of the confidence rating) Under* *Assumptions 1–4*,

$$\text{info}=I\left(Y;\hat{Y},C\right)=E\left[pti\left(C\right)\right].$$

See supplement for the proof. Continuing the example in Figure 1, the confidence ratings *c* = 60% and *c* = 90% in Figure 1a translate into predictionwise transmitted information pti(60%) = 0.03 bit and pti(90%) = 0.53 bit as shown in Figure 1b. Together with the probabilities for both cases (2/3 for low confidence rating and 1/3 for high confidence rating), these yield the transmitted information $\text{info}=E\left[pti\left(C\right)\right]=\frac{2}{3}\cdot 0.03bit+\frac{1}{3}\cdot 0.53bit=0.20bit$ as shown in Figure 1c.

In the following, we will show that accuracy acc and transmitted information info are only loosely related. Knowing the accuracy of a classifier only constrains *E*[*C*] (Figure 1a). But different confidence distributions *f*_*C*_ with the same expected value *E*[*C*] can have different *E*[pti(*C*)] (Figure 1c) due to the non-linearity of pti(*C*). In consequence, two classifiers with the same accuracy can transmit different amounts of information depending on their confidence distribution. Next, we demonstrate this relationship in an example and then present the upper and lower bounds the two performance measures impose on each other.

## A SIGNAL DETECTION THEORY EXAMPLE

**Figure 2.**
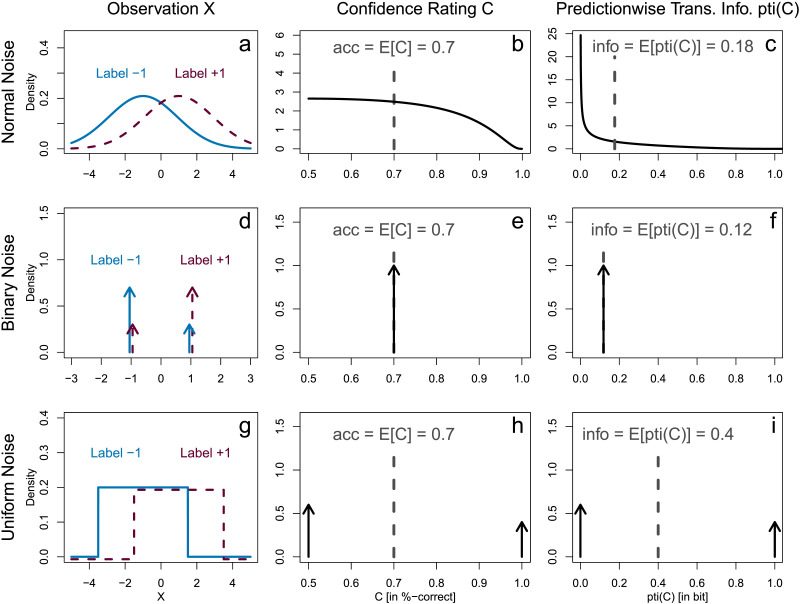
Accuracy and Transmitted Information for Different Noise Distributions. *Note*. Different noise distributions over the observation *X* (left column) induce different confidence distributions (middle column, cf. Figure 1a) as well as different predictionwise transmitted information distributions (right column, cf. Figure 1c). We show three example noise distributions: normal, binary, and uniform noise (each in one row). In all examples, we fixed the accuracy, that is, the expected value of the confidence distribution to acc = *E*[*C*] = 70%. Because the translation from confidence ratings *C* to predictionwise transmitted information pti(*C*) is non-linear (i.e., strictly convex, see Figure 1b), the transmitted information info = *E*[*pti*(*C*)] varies across the three examples.

To see how accuracy and transmitted information are interrelated in our setting, consider an example based on signal detection theory (SDT; Green & Swets, 1988; Wixted, 2020; see Feldman, 2021a, 2021b for an information-theoretic perspective on SDT). In standard SDT, two equally probable labels (*p*_+_ = 0.5) are associated with normal distributions that have the same standard deviation *σ* but different means *μ*_+1_ and *μ*_−1_, see Figure 2a. Their mean difference relative to the standard deviation is the sensitivity $d^{\prime }=\frac{\mu_{+1}-\mu_{-1}}{\sigma }$.

The true label *Y* ∈ {−1, +1} is randomly determined and the classifier gets to see a noisy observation *X* sampled from the corresponding normal distribution with mean *μ_Y_*. Following our idealistic Assumptions 3–4, the classifier knows the posterior probability for the true label given the observation *X*, so that it can make the optimal prediction $\hat{Y}=\arg\max_{y}P\left(Y=y|X\right)$ with confidence rating *C* = max*_y_ P*(*Y* = *y*|*X*) = (1 +*e*^−*d*′∣*X*/*σ*∣^)^−1^.

The sensitivity *d*′ determines the differentiability of observations *X*. In Figure 2, we present an example with sensitivity *d*′ = 1.05. Fixing *d*′ determines the distribution over observations, *f*_*X*_ (Figure 2a), and thereupon the confidence distribution, *f*_*C*_ (Figure 2b). This in turn determines the accuracy which is here acc = *E*[*C*] = 70%. Note that due to Assumption 4, the decision criterion is always set to the optimal threshold maximizing the accuracy and will therefore not play a role in what follows. Further, the confidence distribution translates into a distribution on the predictionwise transmitted information, *f*_pti(*C*)_ (Figure 2c), and therefore also determines the transmitted information which is here info = *E*[pti(*C*)] = 0.18 bit.

The accuracy of acc = 70% does not uniquely determine the confidence distribution and therefore allows for different values of the transmitted information. In the second row of Figure 2, we show another distribution of observations, confidence ratings, and predictionwise transmitted information that produces the same accuracy but a lower transmitted information, info = 0.12 bit. In this row, observations are binary. This corresponds to a situation in which there is no differentiation between high vs. low certainty from observations. This noise distribution is equivalent to the worst case situation, which the established measures of Type 2 performance evaluate as meta–*d*′ = 0 and *M*−ratio = 0 (corresponding to fully overlapping truncated normal distributions) because there is no differentiation between uncertainties from confidence ratings.

In the third row of Figure 2, we show yet another example departing from normal noise. Here, observations are uniformly distributed. With this, classifiers make random guesses with a confidence rating *c* = 50% when an observation falls in the overlap of the two uniform distributions. In contrast, predictions come with perfect certainty *c* = 100% if the observation falls outside the overlap. The uniform distributions are shifted to match the same accuracy as before, acc = 70%. But now the transmitted information is substantially higher than in the two examples before, info = 0.4 bit. Such strictly separated confidence distributions may be implausible for the typical dot motion tasks. But an approximation of this theoretical boundary case can be found in general knowledge question tasks. There, confidence ratings are sometimes bimodally distributed with either very low values when participants are just guessing or very high values when they do know the correct answer without many in-between cases. A particularly clear example of this are Experiments 1a and 1b of Little (2023) with bimodal confidence rating histograms associated with a clear separation between low versus high accuracies.

These examples demonstrate that different confidence distributions (coming for example from different noise distributions underlying observations) can lead to the same overall accuracy but substantially different amounts of transmitted information. However, note that we are not interested in the underlying noise distributions because we make no assumptions on the internal mechanisms or observations *X* on which a classifier bases its predictions—we only presented these cases as instructive examples. Other mechanisms can create the same ambiguity between accuracy and transmitted information and our results hold in general for any such mechanism.

Next, we show that the confidence distributions in Figure 2 are indeed the limiting cases: For a given accuracy, the transmitted information is maximal if confidence distributions are as in the uniform noise case in Figure 2h and minimal if they are as in the binary noise case in Figure 2e.

## ACCURACY-INFORMATION AMBIGUITY

We now present the bounds that the accuracy imposes on the transmitted information. We thereby determine the highest and the lowest possible transmitted information for a classifier with a fixed accuracy. We first present these bounds in the simple case of binary labels and then generalize them to multiple labels.

### Bounds for Binary Labels

**Figure 3.**
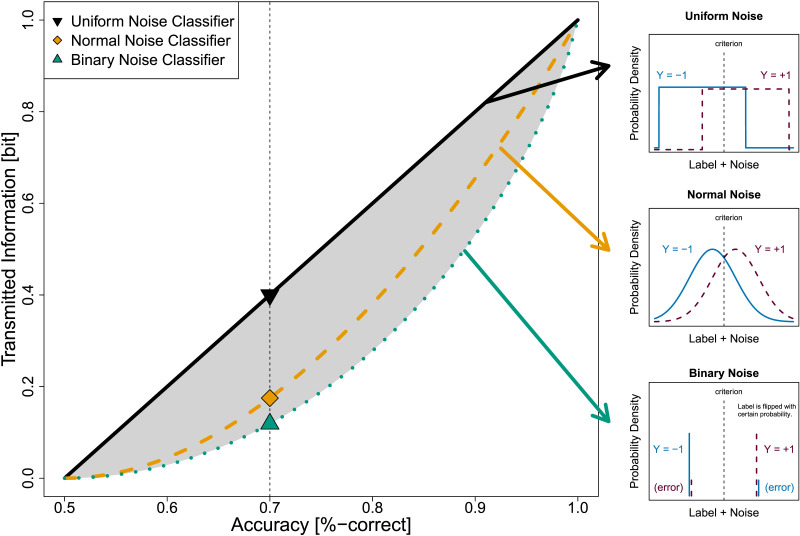
Accuracy Imposes Bounds on Transmitted Information. *Note.* For binary (*L* = 2) and a priori equally probable labels (*p*_+_ = 0.5), any classifiers’ accuracy and transmitted information lie in the gray shaded area: Their accuracy (*x*-axis) imposes upper (black solid line) and lower bounds (green dotted line) on their transmitted information (*y*-axis). The upper bound is attained by underlying uniform noise observations (Figure 2g–i) and the lower bound by binary noise observations (Figure 2d–f). Normal noise observations (Figure 2a–c) are an intermediate case (orange dashed line).

If the label *Y* is binary, the accuracy of a classifier bounds its transmitted information as given by Theorem 3. We visualize this result in Figure 3: Any classifier following our assumptions must lie in the shaded area that represents the loose relationship between accuracy and transmitted information.

**Theorem 3**. *(Binary information bounds) Under* *Assumptions 1–4**, a classifier with known accuracy* acc *but unknown confidence distribution f_C_ has a transmitted information of at most*

$$\max_{f_{C}}\text{info}=H_{2}\left(p_{+}\right)-2\left(1-acc\right)$$

*and at least*

$$\min_{f_{C}}\text{info}=H_{2}\left(p_{+}\right)-H_{2}\left(acc\right).$$

*Proof*.

info = *E*[pti(*C*)] (Prop. 2)

$=E\left[pti\left(\frac{1-C}{1-0.5}\cdot 0.5+\frac{C-0.5}{1-0.5}\cdot 1\right)\right]$  (Split *C* to 0.5 and 1)

$\leq E\left[\frac{1-C}{1-0.5}\cdot \underset{H_{2}\left(p_{+}\right)-1}{\underbrace{pti\left(0.5\right)}}+\frac{C-0.5}{1-0.5}\cdot \underset{H_{2}\left(p_{+}\right)}{\underbrace{pti\left(1\right)}}\right]$ (Convexity of pti)

$=H_{2}\left(p_{+}\right)-\frac{1-E\left[C\right]}{1-0.5}$  (Linearity of *E*)

 = *H*_2_(*p*_+_) − 2(1 − acc) (Prop. 1)

info = *E*[pti(*C*)] (Prop. 2)

≥ pti(*E*[*C*]) (Jensen’s Inequality for convex pti)

 = *H*_2_(*p*_+_) − *H*_2_(acc) (Prop. 1, Def. of pti)

Our proof shows that the key feature affecting the transmitted information is how far confidence ratings are separated. The proof for the upper bound has been presented before by Hu (2014) and the proof for the lower bound is also a corollary of Fano’s inequality (pp. 37–38 in Cover & Thomas, 2006).

The upper bound is attained when maximally distinguishing certain vs uncertain cases. Thus, the upper bound represents classifiers making predictions either as random guesses (*c* = 50%) or with absolute certainty (*c* = 100%) weighted such that the given overall accuracy is preserved. This case has been mentioned as an extreme mixture case by Rahnev and Fleming (2019) and Dayan (2023). The lower bound is attained when there is no differentiation in confidence ratings at all. Thus, in this case, classifiers always make predictions with the same, intermediate confidence rating (which is then equal to the accuracy due to calibration, *c* = acc).

Because the difference between the highest and lowest transmitted information reflects the ability of a classifier to differentiate between different levels of confidence, the transmitted information can be used as a measure of Type 2 performance. But first, we will discuss the relation of these boundary cases to receiver operator characteristic (ROC) curves and a generalization of Theorem 3 to the case of more than two labels, *L* ≥ 2. Readers who are mainly interested in the application of our bounds for metacognition experiments with binary labels may skip ahead to Section Surplus Information as a Type 2 Performance Measure.

### Bounds for Receiver Operator Characteristic (ROC) Curves

The boundary cases described above are also related to the bounds on receiver operator characteristic (ROC) curves (Fawcett, 2006). Two types of ROC curves are typically discussed in metacognition research: Pseudo Type 1 ROC curves and Type 2 ROC curves (e.g., Kellij et al., 2021; Meuwese et al., 2014). Pseudo Type 1 ROC curves consider threshold classifiers on prediction-signed confidence ratings, $\hat{Y}\cdot C$, which act as pseudo observations: Values close to 0 correspond to low confidence and large deviations to either side correspond to high confidence in either prediction. These pseudo observations $\hat{Y}\cdot C$ take the place of observations *X* in regular Type 1 ROC curves (Galvin et al., 2003; Maniscalco & Lau, 2014).

**Figure 4.**
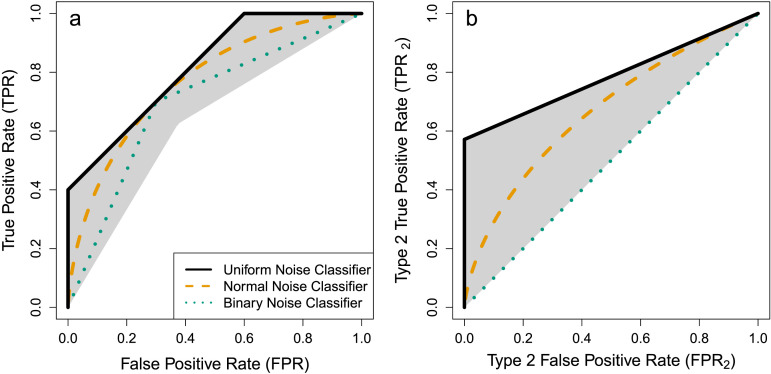
Bounds on Pseudo Type 1 and Type 2 Receiver Operator Characteristic (ROC) Curves. *Note.* (a) Any classifier with a given accuracy (here exemplified again with acc = 70%), has a pseudo Type 1 true positive rate that is bounded by its pseudo Type 1 false positive rate: All pseudo Type 1 ROC curves (under the assumptions of Proposition 4) must lie within the gray area. Colored lines represent the example classifiers from above with underlying uniform, normal, and binary noises. Note that uniform and binary noises are discrete and require randomization to generate the intermediate steps on the ROC curves. (b) Similarly, the Type 2 true positive rate is bounded by a function of the Type 2 false positive rate.

In our setup with perfectly calibrated confidence ratings and when assuming a certain ratio of true labels (for simplicity, we assume *P*(*Y* = +1) = *P*(*Y* = −1) = 0.5), the true positive rate $TPR=P\left(\hat{Y}\cdot C>t|Y=+1\right)$ of a threshold classifier with a given accuracy is bounded by a function of its false positive rates $FPR=P\left(\hat{Y}\cdot C>t|Y=-1\right)$ as given by Proposition 4. These bounds are visualized in Figure 4a.

**Proposition 4**. *(Bounds on pseudo Type 1 ROC curves) Under* *Assumptions 1–4* *and assuming P*(*Y* = +1) = *P*(*Y* = −1) = 0.5*, the true positive rate (TPR) of any classifier with accuracy* acc *is bounded by a function of its false positive rate (FPR)*,

$$\min\left\{\frac{FPR}{2\left(1-acc\right)},2\left(1-acc\right)FPR+\left(2acc-1\right)\right\}\leq TPR\leq FPR+\left(2acc-1\right).$$

See supplement for the proof. As shown in Figure 4a, the uniform noise classifier (highest transmitted information) corresponds to the upper bound; but the binary noise classifier (lowest transmitted information) does not correspond to the lower bound here. This is because the binary noise classifier in our example gives confidence ratings *C* that are independent of the predictions $\hat{Y}$. In contrast, Proposition 4 does not make this independence assumption allowing more leeway and leading to a lower bound (lower edge of the gray area in Figure 4a) below the binary noise classifier (green dotted line). Had we made the independence assumption, the lower bound would correspond to the binary noise classifier.

The second variant are Type 2 ROC curves, for which we replace pseudo observations $\hat{Y}\cdot C$ by confidences *C* as well as *Y* = +1 and *Y* = −1 in the conditionals by $Y=\hat{Y}$ and $Y\neq \hat{Y}$ reflecting how confidence ratings are given in correct versus incorrect predictions (Clarke et al., 1959; Kanai et al., 2010). Similar to pseudo Type 1 ROC curves, thresholds on confidence ratings then result in a Type 2 true positive rate ${TPR}_{2}=P\left(C>t|\hat{Y}=Y\right)$ and a Type 2 false positive rate ${FPR}_{2}=P\left(C>t|\hat{Y}\neq Y\right)$. Again, we find bounds for these Type 2 ROC curves as visualized in Figure 4b. As expected, these bounds coincide with the boundary cases from our previous results and the underlying normal noise case is an intermediate case.

**Proposition 5**. *(Bounds on Type 2 ROC curves) Under* *Assumptions 1–4* *and assuming P*(*Y* = +1) = *P*(*Y* = −1) = 0.5*, the Type 2 true positive rate* TPR_2_ *of any classifier with accuracy* acc *is bounded by its Type 2 false positive rate* FPR_2_,

$${FPR}_{2}\leq {TPR}_{2}\leq 1-\frac{1-acc}{acc}\left(1-{FPR}_{2}\right).$$

See supplement for the proof.

### Bounds for Finite Number of Labels

We generalize the bounds in Theorem 3 to classification settings with arbitrary (but finitely) many labels. For this, we need to adapt our assumptions. We denote the number of labels by *L*. We again assume that the prior probabilities of these labels are known.

**Assumption 5** (Discrete Labels). *y* ∈ {1, …, *L*} *with L* ∈ ℕ *and L* ≥ 2

**Assumption 6** (Known Prior). ∀*y* ∈ {1, …, *L*}:*p*_*y*_ = *P*(*Y* = *y*)

A classifier now outputs a prediction $\hat{Y}$ together with a confidence vector *C* = (*C*_1_, …, *C*_*L*_)*^T^* that indicates the posterior probability for each label given the response. We again assume that these confidence ratings are perfectly calibrated. This assumption is called *full calibration* (Kull et al., 2019; Lin et al., 2022). As an alternative, we could have assumed that only the posterior probability for the predicted label is known but not the probabilities for the non-predicted labels. We omit the case of this alternative assumption here. Instead we assume that a confidence vector indicates the posterior probability for each label and, additionally, that classifiers predict the most probable label.

**Assumption 7** (Perfect Calibration). *c*_*y*_ = *P*(*Y* = *y* |*C* = *c*)

**Assumption 8** (Bayes Predictions). $\hat{Y}=\arg\max_{y}C_{y}$

We also need to update Propositions 1 and 2 for relating confidence vectors to accuracy and transmitted information. This is straightforward and we defer the details to the supplement. We can then again provide tight bounds on the transmitted information given the accuracy of a classifier.

**Theorem 6**. *(Discrete information bounds) Under* *Assumptions 5–8**, a classifier with known accuracy* acc *but unknown confidence distribution f_C_ has a transmitted information of at most*

$$\max_{f_{C}}\text{info}=\left(\sum_{l=1}^{L}p_{l}\log\left(\frac{1}{p_{l}}\right)\right)-\frac{\left(\frac{1}{m_{1}}-acc\right)\log\left(m_{2}\right)+\left(acc-\frac{1}{m_{2}}\right)\log\left(m_{1}\right)}{\frac{1}{m_{1}}-\frac{1}{m_{2}}}$$

where *m*_1_ = ⌊1/acc⌋ and *m*_2_ = ⌊1/acc⌋ + 1. Its transmitted information is at least

$$\min_{f_{C}}\text{info}=\left(\sum_{l=1}^{m_{3}}p_{l}\log\left(\frac{1}{p_{l}}\right)\right)-acc\log\left(\frac{1}{acc}\right)-\left(q-acc\right)\log\left(\frac{m_{3}-1}{q-acc}\right)$$

*where m*_3_ *be the largest number in* 1, …, *L such that* $p_{m_{3}}\geq \frac{\left(\sum_{l=1}^{m_{3}}p_{l}\right)-acc}{m_{3}-1}$ *and* $q=\sum_{l=1}^{m_{3}}p_{l}$.

See supplement for the proof. The upper and lower bounds have been proven before by Berger (2003) and Hledík et al. (2019), respectively, and we add our simplified variants of these proofs here.

In this setting, a classifier attains the highest information when it always excludes all but *m*_1_ or *m*_2_ alternatives while giving these alternatives equal confidence ratings. For example, when predicting *L* = 4 labels with equal prior probabilities and an accuracy acc = 0.4, the classifier would ideally restrict the possible labels to *m*_1_ = ⌊1/0.4⌋ = ⌊2.5⌋ = 2 or *m*_2_ = 3 labels resulting in responses like “The true label is *Y* = 1 or *Y* = 2” or “The true label is *Y* = 1, *Y* = 3, or *Y* = 4” with different combinations of these labels. These two cases, excluding all but 2 or all but 3 labels, are then weighted to yield the accuracy of 40%. Interestingly, this corresponds to an unexpected generalization of the *L* = 2 case: Instead of always predicting either as a random guess (25% for *L* = 4) or with absolute certainty (100%), the best case classifier here excludes as many labels as possible with absolute certainty while remaining equally uncertain about the rest. This exclude-all-but-*m*_1_-or-*m*_2_-labels strategy maximizes the transmitted information for a given accuracy.

The lowest information is attained by classifiers always predicting one label with a confidence rating equal to the overall accuracy and giving uniform confidence ratings to the remaining labels. Continuing the previous example, the worst classifier may respond: “The true label is *Y* = 3 with probability 40%, or any of the other three labels each with a probability of 20%”. If there are labels that are too rare to fit into this scheme, the worst case classifier always returns their prior probability. For example, when the prior probabilities are *p* = (32%, 32%, 32%, 4%)*^T^*, the worst case classifier would produce responses like “The true label is *Y* = 1 with probability 40%, *Y* = 2 or *Y* = 3 each with probability 28%, or as always *Y* = 4 with probability 4%”.

With these bounds in place, we now construct a measure for the Type 2 performance of a classifier.

## SURPLUS INFORMATION AS A TYPE 2 PERFORMANCE MEASURE

Because the transmitted information is sensitive to the uncertainty differentiation, it captures a classifier’s Type 2 performance. Based on this observation, Dayan (2023) has recently suggested information-theoretic measures of Type 2 performance (see also a short mention in Rausch & Zehetleitner, 2019). We add a new suggestion here, which we dub the relative metainformation, RMI. Using the information-theoretic bounds we discussed above allows us to keep the range of possible RMI values constant for different Type 1 performances of a classifier. In contrast, other established Type 2 measures can take values in different ranges depending on the Type 1 performance, which constitutes an undesirable confound. Our measure makes a step towards decoupling Type 1 and Type 2 performance measures.

### Surplus Information as Metainformation

While the accuracy of a classifier measures purely Type 1 performance, the transmitted information incorporates both, Type 1 *and* Type 2 performance. This is apparent from the (loose) relationship between accuracy acc and information $\text{info}=I\left(Y;\hat{Y},C\right)$ as shown before in Figure 3. To account for the Type 1 performance, we subtract the lowest possible transmitted information for the observed accuracy. This leaves the *surplus transmitted information*: info − min info(acc).

**Figure 5.**
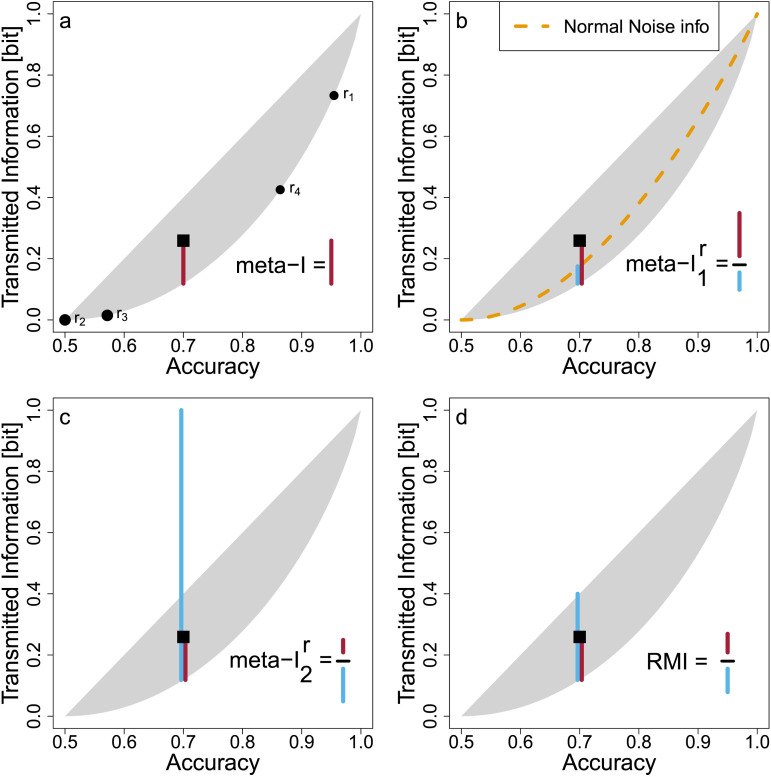
Estimation of the Four Information-Theoretic Measures of Type 2 Performance. *Note.* (a) For each response in the example data from Table 1, the black dots show the estimated accuracy or calibrated confidence (*x*-axis) and the transmitted information (*y*-axis). Their weighted mean (black square) reflects the overall accuracy and information. The distance between the overall transmitted information from the lower bound is the first information-theoretic measure, $\hat{meta}-I$ (red line). (b) The second information-theoretic measure, $\hat{meta}-I$, normalizes $\hat{meta}-I$ by the value that would have been produced by a normal noise classifier (orange dashed line) with the same Type 1 performance (blue line). (c) The third measure, $\hat{meta}-I_{2}^{r}$, instead normalizes by the total amount of remaining uncertainty given the accuracy (blue line). (d) Our fourth measure, $R\hat{M}I$, normalizes by the range of possible values that $\hat{meta}-I$ can take according to Theorem 3 (blue line).

Dayan (2023) approached the situation in a different way. He set out to quantify the information that confidence ratings transmit about the accuracy and dubbed this the metainformation, $\text{meta}-I=I\left(Y\cdot \hat{Y};C\right)$. We show here, that both approaches are equivalent, meta−ℐ = info − min info(acc). This is because the transmitted information can be partitioned, $I\left(Y;\hat{Y},C\right)=I\left(Y;\hat{Y}\right)+I\left(Y\cdot \hat{Y};C\right)$, where the first part is completely determined by the accuracy, $I\left(Y;\hat{Y}\right)=\min\text{info}\left(acc\right)$, and the second part corresponds to the Type 2 information from the confidence ratings, $\text{meta}-I=I\left(Y\cdot \hat{Y};C\right)$. Thus, quantifying the information that confidence ratings transmit about the accuracy is equivalent to our perspective of the surplus information. We stick with the label meta−ℐ for consistency but follow the perspective of surplus information because it will give rise to a natural normalization (see Figure 5d).

**Proposition 7**. *(Surplus information is equal to metainformation) Under Assumptions 1–4, the metainformation*, $\text{meta}-I=I\left(Y\cdot \hat{Y};C\right)$, *is equal to the surplus information, meta−ℐ = info − min info(acc).*

*Proof*. We note $H\left(Y|\hat{Y}\right)=H\left(Y\cdot \hat{Y}\right)=H_{2}\left(acc\right)$ because both random variables follow a Bernoulli distribution with probability acc.

$$P\left(Y=+\hat{Y}|\hat{Y}\right)=acc\;P\left(Y\cdot \hat{Y}=+1\right)=acc$$

$$P\left(Y=-\hat{Y}|\hat{Y}\right)=1-acc\;P\left(Y\cdot \hat{Y}=-1\right)=1-acc$$

Similarly, $H\left(Y|\hat{Y},C\right)=H\left(Y\cdot \hat{Y}|C\right)=H_{2}\left(C\right)$ but with Bernoulli probability *C* instead of acc.

$$P\left(Y=+\hat{Y}|\hat{Y},C\right)=C\;P\left(Y\cdot \hat{Y}=+1|C\right)=C$$

$$P\left(Y=-\hat{Y}|\hat{Y},C\right)=1-C\;P\left(Y\cdot \hat{Y}=-1|C\right)=1-C$$

With this, we can decompose the transmitted information as desired.

$$\begin{array}{l} \text{info}=I\left(Y;\hat{Y},C\right)\\ =H\left(Y\right)-H\left(Y|\hat{Y},C\right)\\ =H\left(Y\right)\underset{=0}{\underbrace{-H\left(Y|\hat{Y}\right)+H\left(Y\cdot \hat{Y}\right)}}-H\left(Y\cdot \hat{Y}|C\right)\\ =H_{2}\left(p_{+}\right)-H_{2}\left(acc\right)+I\left(Y\cdot \hat{Y};C\right)\\ =\min\text{info}\left(acc\right)+\text{meta}-I\\ \Leftrightarrow \text{meta}-I=\text{info}-\min\text{info}\left(acc\right) \end{array}$$

### Example Computation of Existing Information-Theoretic Measures

In the following, we will discuss multiple information-theoretic measures of Type 2 performance that are based on the surplus information. The difference between these measures lies in the different ways they are normalized. In addition to the three existing information-theoretic measures, meta−ℐ, $\text{meta}-I_{1}^{r}$ and $\text{meta}-I_{2}^{r}$, we add our new suggestion, the relative metainformation, RMI. Our measure normalizes the surplus information using the lower and upper bounds on the transmitted information discussed above. For the purpose of comparison, we will also shortly present the established SDT-based measures, meta−*d*′ and *M*−ratio.

(a)  meta−*d*′: sensitivity of the SDT model that best fits confidence ratings conditioned on responses
(b)  $M-\text{ratio}=\frac{\text{meta}-d^{\prime }}{d^{\prime }}$: the metasensitivity relative to the (regular) sensitivity
(c)  meta−ℐ = info − info_lower_(acc): the metainformation or surplus information
(d)  $\text{meta}-I_{1}^{r}=\frac{\text{meta}-I}{\text{meta}-I\left(d^{\prime }\right)}$: the metainformation relative to the normal noise case
(e)  $\text{meta}-I_{2}^{r}=\frac{\text{meta}-I}{H\left(Y=\hat{Y}\right)}$: the metainformation relative to the total amount of remaining uncertainty given the accuracy
(f)  $RMI=\frac{\text{meta}-I}{\max\text{info}\left(acc\right)-\min\text{info}\left(acc\right)}$: the metainformation relative to the admissible range

To demonstrate how to estimate these measures, we introduce example data in Tables 1–2 and visualize these measures in Figure 5. Table 1 shows the absolute frequencies of observed combinations between true labels and a classifier’s responses. For example, the classifier gave the first out of four responses (*r*_1_) 88 times out of which 84 were given when the stimulus category was *Y* = −1 and four when the other stimulus category was *Y* = +1, corresponding to a prediction for $\hat{Y}=-1$ with high confidence of around 84/88 = 95%. There were 200 trials for each stimulus category (row sums) for a total of 400 observed responses. Continuing the examples from above, we kept accuracy in this example data at acc = 70% and equivalently, the sensitivity at *d*′ = 1.05. Regarding SDT-based measures, this data set yields meta−*d*′ = 1.9 and *M*−ratio = meta−*d*′/*d*′ = 1.8 indicating better Type 2 performance than underlying normal noise would have produced.

**Table 1.** Example of Observed Absolute Frequencies

True label	Response			
*Y*	*r*_1_	*r*_2_	*r*_3_	*r*_4_
−1	84	56	48	12
+1	04	56	64	76

*Note.* For a fictitious participant, counts of each combination of true label *Y* (presented stimulus category) and response *R* are shown. For example, of the 200 trials with label *Y* = −1 (first row), 84 received an *r*_1_ response (first column).

**Table 2.** Example of Observed Relative Frequencies

True label	Response			
*Y*	*r*_1_	*r*_2_	*r*_3_	*r*_4_
−1	0.21	0.14	0.12	0.03
+1	0.01	0.14	0.16	0.19
${\hat{m}}_{i}$	0.22	0.28	0.28	0.22
${\hat{c}}_{i}$	0.95	0.50	0.57	0.86
$pti\left({\hat{c}}_{i}\right)$	0.73	0.00	0.01	0.43

*Note.* For the count data from Table 1, relative frequencies (count divided by the total number of counts) are shown. For each response *r*_*i*_, the columnwise sum of frequencies yields the margin ${\hat{m}}_{i}$, the ratio determines the confidence rating ${\hat{c}}_{i}$ which in turn determines the predictionwise (i.e., responsewise) transmitted information $pti\left({\hat{c}}_{i}\right)$.

We divide these absolute frequencies by the total number of observations, which yields the maximum likelihood estimate of the joint probability distribution in Table 2. From this, we estimate marginal frequencies, accuracies, and information for each response (estimates are denoted by hats): The estimated marginal probabilities ${\hat{m}}_{i}$ are the columnwise sums of the relative frequencies. The estimated confidence ratings ${\hat{c}}_{i}$ are the highest relative frequency per column divided by the marginals ${\hat{m}}_{i}$. This follows from Assumption 4 ensuring that confidence ratings are above 50%. It also removes the strong Assumption 3 (perfect calibration) from the equation because confidence ratings are estimated empirically rather than relying on the classifier’s ratings. See the discussion for the implications of this approach. From these estimated confidence ratings, the predictionwise transmitted information is estimated to be $pti\left({\hat{c}}_{i}\right)$. Thus, each response *i* is associated with a weight ${\hat{m}}_{i}$, a confidence ratings ${\hat{c}}_{i}$ indicating its conditional accuracy, and a transmitted information $pti\left({\hat{c}}_{i}\right)$. These values are visualized as black circles along the lower bound of the gray shape in Figure 5a.

From ${\hat{m}}_{i}$, ${\hat{c}}_{i}$, and $pti\left({\hat{c}}_{i}\right)$ of each response *i*, we estimate the overall accuracy $a\hat{c}c$ and transmitted information $\text{in}\hat{f}o$. Following Propositions 1 and 2, these are the weighted means: The overall accuracy is the weighted mean of confidence ratings, $a\hat{c}c=\sum_{i}{\hat{c}}_{i}\cdot {\hat{m}}_{i}=0.7$, and the transmitted information the weighted mean of predictionwise transmitted information, $\text{in}\hat{f}o=\sum_{i}pti\left({\hat{c}}_{i}\right)\cdot {\hat{m}}_{i}=0.26$. Figure 5 provides an intuitive understanding of the information-theoretic measures: Individual responses (black circles along the lower bound) determine their center of gravity (square) reflecting the accuracy $a\hat{c}c$ as the weighted mean along the *x*-axis and the information $\text{in}\hat{f}o$ as the weighted mean along the *y*-axis.

To contextualize the estimated transmitted information $\text{in}\hat{f}o$, we now consider its lower bound given the estimated accuracy $a\hat{c}c$. For an accuracy $a\hat{c}c=0.7$ and prior *p*_+_ = 0.5, Theorem 3 yields the lowest possible transmitted information of $\min\text{info}\left(a\hat{c}c\right)=0.12\;bit$. With that, the surplus information is

$$\hat{meta}-I=\hat{info}-\min\text{info}\left(a\hat{c}c\right)$$

=0.26−0.12=0.14bit.

In Figure 5a, this value is visualized as the distance of the square from the lower bound (red line).

Next to meta−ℐ, Dayan (2023) suggested two more measures with different normalizations. The first normalized information-theoretic measure, $\text{meta}-I_{1}^{r}$ (see Figure 5b), considers meta−ℐ relative to what would have been produced by a normal noise classifier with the same Type 1 sensitivity, meta−ℐ(*d*′). Here, the sensitivity *d*′ = 1.05 determines the normalizer meta−ℐ(*d*′) = 0.056 bit leading to the measure

$$\hat{meta}-I_{1}^{r}=\frac{\hat{meta}-I}{\text{meta}-I\left(d^{\prime }\right)}$$

$$=\frac{0.14}{0.056}=2.5.$$

Thus, the observed confidence ratings transmit 2.5 times more metainformation than an observer with the same Type 1 performance but underlying normal noise. This measure is similar to *M*−ratio = meta−*d*′/*d*′, which also normalizes by the sensitivity *d*′. Both measures aim to produce constant values, $\text{meta}-I_{1}^{r}=1$ and *M*−ratio = 1, whenever the underlying noise indeed follows a normal distribution.

The third information-theoretic measure normalizes meta−ℐ by the total amount of remaining uncertainty, $\text{meta}-I_{2}^{r}$ (see Figure 5c). The remaining uncertainty is here $H\left(Y=\hat{Y}\right)$, which leads to the measure

$$\hat{meta}-I_{2}^{r}=\frac{\text{meta}-I}{H\left(Y=\hat{Y}\right)}$$

$$=\frac{0.14}{0.88}=0.16.$$

But note that in our setting, Theorem 3 prohibits any classifier from reducing the remaining uncertainty entirely via confidence ratings. This is because $\text{meta}-I_{2}^{r}=1$ would imply $H\left(Y=\hat{Y}\right)-H\left(Y\cdot \hat{Y}|C\right)=H\left(Y=\hat{Y}\right)$ and $H\left(Y\cdot \hat{Y}|C\right)=0$, requiring predictions to be made exclusively with *C* = 100% confidence ratings. No classifier with an accuracy below acc = 100% can fulfill this requirement under our Assumption 4 or 8, which require optimally coded predictions. Thus, the normalizer $H\left(Y=\hat{Y}\right)$ is overly penalizing in our setting. In contrast, Dayan (2023) suggested this normalization for potentially suboptimally coded predictions. If this is allowed, the upper bound of the transmitted information is trivially given by the entire entropy *H*(*Y*) and independent of the classifier’s accuracy. We argue in the discussion that Assumptions 4 and 8 are desirable because they mitigate the confound between Type 1 and Type 2 measures.

### Our New Information-Theoretic Measure: Relative Metainformation

Inspection of the three existing measures in Figure 5a–c, suggests that one relevant normalization of the metainformation is missing from the current measures: one that incorporates the upper and lower bounds of the admissible range of information values for normalization as in Figure 5d. This makes additional use of the upper bound, maxinfo(acc) from Theorems 3 and 6 and leads to a well-scaled measure of Type 2 performance, which we call the relative metainformation, RMI:

$$\text{Relative}\;\text{Metainformation},\;RMI=\frac{\text{info}-\min\text{info}\left(acc\right)}{\max\text{info}\left(acc\right)-\min\text{info}\left(acc\right)}.$$

RMI values range from 0 to 1 where 0 corresponds to the lowest possible meta−ℐ in the binary noise case (all confidence ratings are on average) and 1 corresponds to the highest possible meta−ℐ in the uniform noise case (all confidence ratings are either 50% or 100%).

In the example data of Table 1 and 2, the accuracy $a\hat{c}c=0.7$ implies that $\text{in}\hat{f}o$ must lie between 0.12 (lower bound) and 0.4 (upper bound, as in Figures 2 and 3). This yields an estimated RMI of

$$R\hat{M}I=\frac{\text{in}\hat{f}o-\min\text{info}\left(a\hat{c}c\right)}{\max\text{info}\left(a\hat{c}c\right)-\min\text{info}\left(a\hat{c}c\right)}$$

$$=\frac{0.26-0.12}{0.4-0.12}=\frac{0.14}{0.28}=0.50,$$

indicating that around half of the possible metainformation for the given accuracy is exhausted by the classifiers’ confidence ratings. One advantage of RMI is that it always takes values in the range from 0 and 1 regardless of the accuracy (Type 1 performance), which is not the case for the other measures as we will show next.

### Comparison of Type 2 Performance Measures: Setup

**Figure 6.**
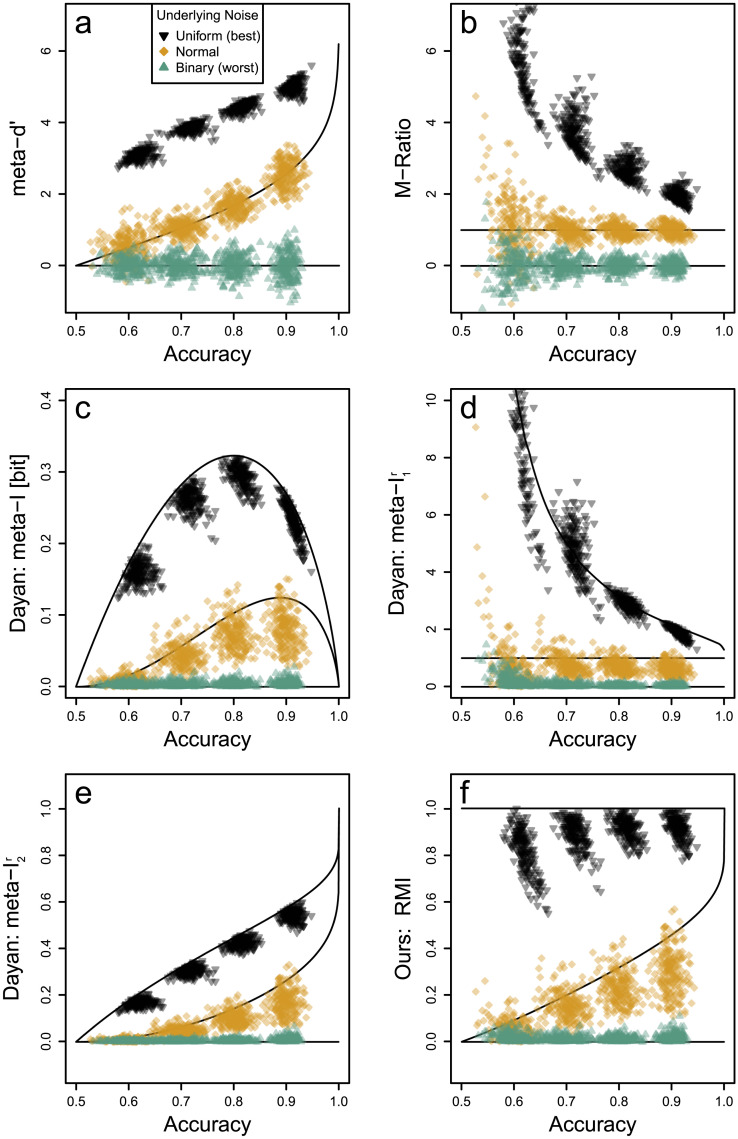
Measures of Type 2 Performance in Comparison. *Note*. We show different measures of Type 2 performance evaluated on simulated data from different noise distributions (uniform, normal, and binary) and accuracies (60%, 70%, 80% and 90%). Black inverted triangles represent simulations from the highest information classifiers, orange diamonds represent normal noise classifiers, and green triangles represent the lowest information classifiers. Lines represent theoretically to-be-expected values (e.g., with infinitely many samples). For all Type 2 measures, except our RMI in subplot f, the range of possible values depends on the Type 1 performances (accuracies on the *x*-axis) revealing a problematic, conceptual confound.

We now demonstrate how different measures of Type 2 performance behave in comparison to each other. We consider six measures of Type 2 performance: two established SDT-based measures, meta−*d*′ and *M*−ratio (Fleming & Lau, 2014; Katyal & Fleming, 2024; Maniscalco & Lau, 2012) as well as the discussed information-theoretic measures, meta−ℐ, $\text{meta}-I_{1}^{r}$ and $\text{meta}-I_{2}^{r}$ from Dayan (2023) and our relative metainformation, RMI. Crucially, only our suggested measure (RMI in Figure 6f) takes values in a constant range independent of the accuracy of the classifier. Therefore, it measures Type 2 performance decoupled from the Type 1 performance to some degree (but also not entirely, see discussion). Nevertheless, it is important to take Type 1 performance measures into account when interpreting Type 2 performance measures and this still applies for RMI.

We simulate data with the three relevant underlying noise distributions discussed earlier: uniform, normal, and binary noise (cf. Figure 2). We vary the accuracy of these distributions in four levels: acc ∈ {60%, 70%, 80%, 90%}. This leads to 12 noise × accuracy conditions. For each condition, we simulate 200 data sets. Each simulated data set is generated by sampling 400 observations (200 from each of the two true labels), see supplement for simulations with more observations. Each observation determines the prediction and calibrated confidence rating based on the respective noise distribution. To emulate a somewhat realistic data set, we bin these confidence ratings into two bins: a low confidence bin *c* ∈ [50%, 75%) and a high confidence bin *c* ∈ [75%, 100%]. For binary noise, we randomly assign each observation into one of the two bins with equal probabilities reflecting the inability of a classifier to differentiate between low vs. high confidence ratings. This produces data as for example shown in Table 1. Note that the binning reduces the transmitted information (Dayan, 2023) and different binnings can lead to different reductions. For simplicity, we stick to this one binning scheme here.

We then applied the six measures mentioned above to this simulated data. For computing the two SDT-based measures, we use the implementation by Rausch and Hellmann (2023). For validation, we also used the two implementations by Craddock (2021) and Lee (2019). All results yielded qualitatively similar results (slight deviations occur due to the implementation of the estimation strategies). There also exist other SDT-based measures (Clarke et al., 1959; Fleming & Lau, 2014) but we will restrict our analysis to the two primary SDT-based measures. The information-theoretic measures were implemented by us.

### Comparison of Type 2 Performance Measures: Evaluation

Our simulation results are shown in Figure 6: Each subplot represents one of the Type 2 performance measures on the *y*-axis plotted against the Type 1 performance (accuracy) on the *x*-axis. In each subplot, each individual point represents one simulated data set. It is evident that all measures agree on the ordering of the noise distributions: Uniform noise is evaluated as the best Type 2 performance while binary noise is the worst with normal noise being an intermediate case.

But while the existing measures (Figure 6a–e) consistently assign values around 0 to the worst case (green triangles), the range of possible values changes because the highest values change for different accuracies. This is an undesirable conceptual confound between what these measures ought to quantify, the Type 2 performance, and the Type 1 performance. The measures meta−*d*′, meta−ℐ, and $\text{meta}-I_{2}^{r}$ show clear dependencies even for the normal noise case. The measures that normalize relative to the normal noise case, *M*−ratio and $\text{meta}-I_{1}^{r}$, achieve some degree of independence from accuracies (Barrett et al., 2013; Guggenmos, 2021, 2022) but transfer the problematic confound to the uniform noise case. (Note that $\text{meta}-I_{1}^{r}$ represented by orange diamonds consistently lies below 1 due to the binning that reduces information.)

In contrast, only our measure (Figure 6f) incorporates the lower and upper bound so that the measured values fall in a constant range: Values close to 0 indicate the lowest possible Type 2 performance, and values close to 1 indicate the highest possible Type 2 performance independent of the accuracy. Nevertheless, it still incurs estimation confounds (as do all the other measures). This can be seen by the slanted point clouds. For example, in Figure 6f, the black inverted triangle clouds all slant downwards: For a fixed classifier, overestimating the Type 1 performance comes with underestimating its Type 2 performance, and vice versa. Moreover, the efficiency of Type 2 performance measures depends on the Type 1 performance (different variabilities along the *y*-axis of the point clouds depending on the accuracy). This points to the inherent difficulties in estimating Type 2 performance independently of Type 1 performance.

### Bias Reduction

Note that the information-theoretic measures of Type 2 performance Figure 6c–f exhibit biases moving their expected values (center of the point clouds) away from the ideal values (lines). This problem arises for information-theoretic measures but not for SDT-based measures because, without distributional assumptions, the transmitted information is notoriously difficult to estimate: The estimation bias can only be mitigated (Archer et al., 2014; Jiao et al., 2017) but not removed entirely (Paninski, 2003).

Here, we suggest a pragmatic approach to reduce the bias of information-theoretic measures: We estimate the bias of the plug-in estimators from above via Monte Carlo simulations based on the observed distribution. That is, when assuming the observed frequencies (e.g., Table 2) to be the true probability distribution, we simulate many data sets and each time compute the respective estimator. This yields an estimated bias that we then subtract. For example, we compute $R\hat{M}I$ from the observed frequency distribution and then sample with replacement, say, *m* = 1,000 times computing a new $R\hat{M}I_{i}^{MC}$. The deviation of the mean of these Monte Carlo simulations from the original estimate determines the estimated bias, $B\hat{i}as=\left(\frac{1}{m}\sum_{i}R\hat{M}I_{i}^{MC}\right)-R\hat{M}I$, which we then subtract from the original estimate, $R\hat{M}I^{\text{bias reduced}}=R\hat{M}I-B\hat{i}as$. This method does not entirely remove bias (which is impossible as noted above) but it makes a compromise between simplicity and effectiveness in bias reduction for a typical metacognition experiment. More advanced bias reduction methods (Archer et al., 2014; Jiao et al., 2017) did not strictly outperform this approach in our simulations.

**Figure 7.**
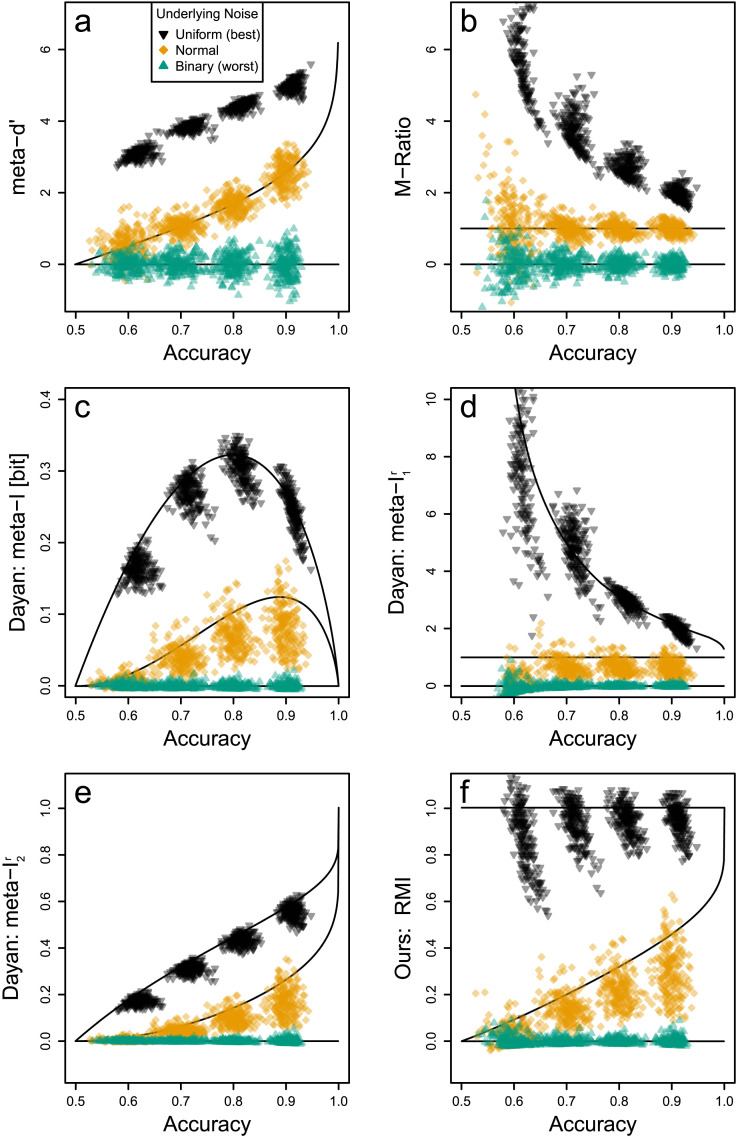
Measures of Type 2 Performance With Bias Reduction in Comparison. *Note*. Same as Figure 6 but with bias reduction applied to the information-theoretic measures in subplots c–f. This brings these measures closer to the ideal values (lines) but not perfectly because bias cannot be entirely removed.

We applied this bias correction to the four information-theoretic measures and obtained an updated Figure 7, which is similar to Figure 6: Subplots a and b remained the same (no bias correction was necessary here) but subplots c–f were bias reduced bringing estimates closer to the ideal lines. Note that this allows information-theoretic estimates to become negative. In particular, $R\hat{M}I^{\text{bias reduced}}$ can take values below 0 and above 1, which is a necessary consequence of bias removal so that the expected values (centers of points clouds) approach the boundaries.

## RELATIVE METAINFORMATION IMPROVES GROUP ACCURACY

The accuracy-information ambiguity we discussed above becomes particularly relevant in group decisions where (individual) Type 2 performance translates into (group) Type 1 performance. We formalize the relation between the Type 2 performance of individual classifiers and a group’s Type 1 performance by presenting new bounds on the group accuracy. We show that the highest vs. lowest group accuracy is attained when individual classifiers with a fixed accuracy have maximal (RMI = 1) vs. minimal Type 2 performance (RMI = 0).

These theoretical results become relevant whenever combining multiple classifiers into a group as is common practice to achieve better predictions. This has been studied in groups of human raters (Bang & Frith, 2017; Einhorn et al., 1977; Koriat, 2015) where groups of medical decision makers have become of particular interest (Barnett et al., 2019; Blanchard et al., 2024; Hasan et al., 2024; Kurvers et al., 2016; and others). Frith and Frith (2012; Frith, 2012) even suggest that a main purpose of explicitly reporting confidence ratings is for collaboration with other humans. Moreover, forming groups of algorithmic classifiers (often called ensembles) is also ubiquitous in the machine learning literature (e.g., Okun et al., 2011; Schapire & Freund, 2013, Chapter 9). Often, multiple artificial neural network classifiers are combined and their predictions aggregated (Ganaie et al., 2022; Hansen & Salamon, 1990). In other cases, algorithmic classifiers have built-in ensembles (Rokach, 2010): For example, random forests consist of multiple tree-classifiers (Akash et al., 2019; Bostrom, 2007; Breiman, 2001; Jiang et al., 2012; Tóth & Pataki, 2008; Winham et al., 2013) and large artificial neural networks can be considered as implementing multiple parallel solutions pathways that are combined in the final layers when using implicit ensemble strategies (Bernstein et al., 2021; Bernstein, 2023; Ganaie et al., 2022). Another application in the machine learning contexts is that of feature selection, where “groups” of features are chosen to make predictions (Epstein et al., 2023; Rong et al., 2022; Sheikhi & Altınçay, 2020; Vergara & Estévez, 2014; Wang et al., 2017).

In all these cases, individual predictions can be weighted by their associated confidence ratings. This is often done (e.g., random forests weigh individual tree predictions by their predictionwise confidence rating, see Akash et al., 2019) and the individual Type 2 performance then becomes of immediate practical relevance: If individual classifiers can differentiate well between certain vs. uncertain predictions, the group can weigh the individual predictions appropriately to achieve better performance. In the machine learning literature, this has been discussed in the margins theory (Bartlett et al., 1998; Biggs et al., 2022): Margins refer to the distances of observations from a classifier’s decision boundary and relate to the notion of confidence (larger margin correspond to higher confidence). Increasing the Type 2 performance (“boosting the margins”) has been shown to improve a group’s performance—even when the individual classifiers’ accuracies remain constant.

Despite the widespread use of this approach, tight bounds on the group accuracy have, to our knowledge, not yet been derived. We close this gap here. For that, we shortly recapitulate the traditional (and known) results from majority voting (MV) and confidence weighted majority voting (CWMV; see also Kuncheva & Rodríguez, 2014). Both use restrictive settings that allow determining the group accuracy uniquely from the accuracy of the individual classifiers but do not fit the notion of weighing predictions by their predictionwise confidence ratings (CWMV gives weights according to the overall accuracy). Then, we introduce the more general setting that we dub *predictionwise confidence weighted majority voting* (PCWMV). In PCWMV, the group accuracy is no longer uniquely determined by the accuracies of the individual classifiers but, instead, substantially depends on the individual Type 2 performances.

### Majority Voting (MV)

In traditional MV (De Condorcet et al., 2014; Grofman et al., 1983; Nitzan & Paroush, 1982), there are *K* classifiers giving predictions ${\hat{Y}}_{1}$, …, ${\hat{Y}}_{K}$ for a true label *Y*. MV uses several simplifying assumptions (due to its historical context in which predictions were votes in a democratic election): All classifiers, here indexed by *k* ∈ 1, …, *K*, have the same accuracy, ${acc}_{k}=P\left({\hat{Y}}_{k}=Y\right)$. Additionally, classifiers’ confidence ratings are constant.

**Assumption 9** (Equal Accuracies). acc_1_ = acc_2_ = … = acc*_K_* ≔ acc

**Assumption 10** (Constant confidence ratings). ∀*k* ∈ {1, …, *K*}:*C*_*k*_ = acc*_k_*

Next, the true labels are assumed to be a priori equally probable. Further, the true labels are equally discernible, that is, confidence ratings are independent of the true label, which is trivial given Assumption 10 but it will become relevant for CWMV.

**Assumption 11** (Equal Prior Probabilities). *p*_+_ = 0.5

**Assumption 12** (Independent confidence ratings). *f*_*C*_*k*_∣*Y*_(*c*_*k*_|*y*) = *f_C_k__*(*c*_*k*_)

Additionally, we assume that individual classifiers’ responses (predictions and confidence ratings) are independent when conditioned on the true label (Berend & Kontorovich, 2014; Kuncheva & Rodríguez, 2014; Zhang & Su, 2004). Unconditional independence would be too strong of an assumption because classifiers’ predictions are necessarily related through the true label if they are competent in predicting that label. The assumption we make here, conditional independence, asserts that there are no further dependencies between classifiers beyond their tie to the true label. This is the usual assumption made for groups of independent experts.

**Assumption 13** (Conditionally Independent Responses). $f_{{\hat{Y}}_{1},C_{1},\ldots ,{\hat{Y}}_{K},C_{K}|Y}\left({\hat{y}}_{1},c_{1},\ldots ,{\hat{y}}_{K},c_{K}\;|\;y\right)=f_{{\hat{Y}}_{1},C_{1}|Y}\left({\hat{y}}_{1},c_{1}\;|\;y\right)\cdot \ldots \cdot f_{{\hat{Y}}_{K},C_{K}|Y}\left({\hat{y}}_{K},c_{K}\;|\;y\right)$

In line with Assumption 4 (individual classifiers make optimal predictions), we assume that the group also combines the individual responses optimally into a group prediction ${\hat{Y}}_{\text{group}}$.

**Assumption 14** (Optimal Group Combination). ${\hat{Y}}_{\text{group}}={\arg\max}_{{\hat{y}}_{\text{group}}}P\left(Y={\hat{y}}_{\text{group}}|{\hat{Y}}_{1},C_{1},\ldots ,{\hat{Y}}_{K},C_{K}\right)$

Under Assumptions 1–4 and the additional MV Assumptions 9–14, the optimal group combination strategy is ${\hat{Y}}_{\text{group}}={\hat{Y}}_{MV}=\mathrm{sign}\left(\sum_{k=1}^{K}{\hat{Y}}_{k}\right)$, see Hansen and Salamon (1990), Nitzan and Paroush (1980), or Moore and Shannon (1956). For groups with an odd number of classifiers (evenly numbered groups run into the tie-breaker problem and are often omitted for simplicity), this leads to the MV accuracy ${acc}_{MV}=P\left(Y={\hat{Y}}_{MV}\right)$ of

$${acc}_{MV}=\sum_{i=\left\lfloor K/2\right\rfloor +1}^{K}\left(\begin{array}{c} K\\ k \end{array}\right){acc}^{i}{\left(1-acc\right)}^{\left(K-i\right)}.$$

Note that the MV accuracy is uniquely determined by the accuracies of the individual classifiers, which is here constant at acc*_k_* = acc.

### Confidence Weighted Majority Voting (CWMV)

As a generalization, CWMV allows individual classifiers to be weighted differently dropping Assumption 9. However, they are given constant weights based on their overall accuracy instead of their predictionwise confidence ratings due to Assumption 10. In this generalization under Assumptions 1–4 and 10–14, the optimal group combination strategy is ${\hat{Y}}_{\text{group}}={\hat{Y}}_{\text{CWMV}}=\mathrm{sign}\left(\sum_{k=1}^{K}\log\left(\frac{C_{k}}{1-C_{k}}\right){\hat{Y}}_{k}\right)$, which follows from Ben-Yashar and Nitzan (1997), Nitzan and Paroush (1982), and Grofman et al. (1983). This yields the CWMV accuracy ${acc}_{\text{CWMV}}=P\left(Y={\hat{Y}}_{\text{CWMV}}\right)$ of

$${acc}_{\text{CWMV}}=\frac{1}{2}\sum_{\left({\hat{y}}_{1},\ldots ,{\hat{y}}_{K}\right)\in {\left\{\pm 1\right\}}^{K}}\;\prod_{k=1}^{K}\left({acc}_{k}^{〚{\hat{y}}_{k}=+1〛}{\left(1-{acc}_{k}\right)}^{〚{\hat{y}}_{k}=-1〛}\right),$$

where 〚⋅〛 are Iverson brackets evaluating to 1 if the statement in the brackets is true and to 0 if it is false. Note that, again, the group accuracy is uniquely determined by the individual accuracies, acc*_k_*.

### Predictionwise Confidence Weighted Majority Voting (PCWMV)

Generalizing further, we allow classifiers to give predictionwise confidence ratings. Thereby, we drop Assumption 10. In this case, the optimal group combination strategy remains ${\hat{Y}}_{\text{group}}={\hat{Y}}_{\text{PCWMV}}=\mathrm{sign}\left(\sum_{k=1}^{K}\log\left(\frac{C_{k}}{1-C_{k}}\right){\hat{Y}}_{k}\right)$ with the only difference being that individual confidence ratings *C*_*k*_ can now vary from prediction to prediction. This is routinely done in practical applications but, to our knowledge, the accuracy of optimally combined groups in this setting has not been derived.

As a minor addition, we also drop Assumptions 11–12. These two assumptions may be suited for metacognition experiments in which the two stimulus categories are shown with equal probabilities and are equally well differentiable. But they are restrictive beyond the laboratory setting. This way, our results apply to cases in which one label occurs more frequently than the other (dropping Assumption 11) and in which one label is better differentiable than the other (dropping Assumption 12, allowing what is referred to as *class-specific recall* in Kuncheva & Rodríguez, 2014). This entails that classifiers’ accuracies can depend on the true label: For *Y* = +1, the conditional accuracy is the true positive rate ${acc}_{k+}=P\left({\hat{Y}}_{k}=+1|Y=+1\right)$ and, for *Y* = −1, it is the true negative rate ${acc}_{k-}=P\left({\hat{Y}}_{k}=-1|Y=-1\right)$. Given these label-specific accuracies, the group accuracy is bounded as follows and the boundary cases are related to the boundary cases of RMI.

**Theorem 8**. *(Group accuracy bounds) Under* *Assumptions 1–4, 13 and 14**, a group of classifiers with known true positive rates* acc_1+_*, …*, acc_*K*+_*, and true negative rates*, acc_1−_*, …*, acc_*K*−_*, but unknown response distributions* $f_{{\hat{Y}}_{1},C_{1}},\ldots ,f_{{\hat{Y}}_{K},C_{K}}$ *has a group accuracy of at most*

$$\max_{f_{{\hat{Y}}_{1},C_{1}},\ldots ,f_{{\hat{Y}}_{K},C_{K}}}{acc}_{\text{PCWMV}}=1-\frac{1}{p_{+}^{K-1}}\prod_{k=1}^{K}\left(1-{acc}_{k}\right)\;$$

where acc*_k_* = *p*_+_ ⋅ acc_*k*+_ + (1 − *p*_+_) ⋅ acc_*k*−_. The group accuracy is at least

$$\min_{f_{{\hat{Y}}_{1},C_{1}},\ldots ,f_{{\hat{Y}}_{K},C_{K}}}{acc}_{\text{PCWMV}}=\sum_{\left(\begin{array}{c} {\hat{y}}_{1}\\ \ldots \\ {\hat{y}}_{K} \end{array}\right)\in {\left\{\pm ,1\right\}}^{K}}\max\left\{\;\begin{array}{l} p_{+}\;\cdot \;\prod_{k=1}^{K}{acc}_{k+}^{〚{\hat{y}}_{k}=+1〛}{\left(1-{acc}_{k+}\right)}^{〚{\hat{y}}_{k}=-1〛}\\ \left(1-p_{+}\right)\;\cdot \;\prod_{k=1}^{K}{\left(1-{acc}_{k-}\right)}^{〚{\hat{y}}_{k}=+1〛}{acc}_{k-}^{〚{\hat{y}}_{k}=-1〛} \end{array}\right\}.$$

Under the additional Assumptions 11 and 12,

1. *the upper bound is attained when all classifiers have* RMI = 1,
2. *the lower bound is attained when all classifiers have* RMI = 0*, and*
3. *the lower bound coincides with CWMV accuracy:* min acc_PCWMV_ = acc_CWMV_.

See supplement for the proof.

Note that the PCWMV group accuracy is not uniquely determined by the individual classifiers’ accuracies anymore. Instead, it depends on the exact confidence distributions of the individual classifiers. The best case group accuracy is realized by a group of classifiers with highest Type 2 performance (RMI = 1) and the worst case group accuracy by a group of classifiers with the lowest Type 2 performance (RMI = 0), where the latter case coincides with traditional CWMV because, there, confidence ratings are kept constant by assumption.

### Specialists Outperform Generalists Even Without Coordination

Without understanding the role of Assumption 13, Theorem 8 may appear deceivingly trivial when considering that individual classifiers can *specialize*. For example, the label *Y* may be accompanied by a covariate *S* ∈ {1, …, *K*} indicating different subcases of the classification task. Then, the *k*-th classifier may be specialized to give predictions with high confidence (*c* = 100%) for *S* = *k* and low confidence (*c* = 50%) otherwise. Naturally, these classifiers would exhibit a high Type 2 performance and groups with such specialization perform best (Ben-Yashar et al., 2012). But this requires a coordination of specializations which is prohibited by Assumption 13 because it would entail that confidence ratings are negatively correlated whereas we assume conditionally independent confidence ratings. Interestingly, even for classifiers that can not coordinate their specializations, specialization is the optimal strategy!

As an illustration for this, consider the following intuitive example: Students are preparing for an exam. In the exam, they will have to answer yes-or-no questions (Assumption 1) with both alternatives being a priori equally probable (Assumption 2, 11, and 12). On the day of the exam, the students will be randomly assigned into groups. In these groups, they give their best guess and can communicate their (perfectly calibrated) confidence ratings (Assumption 4 and 3) which are then optimally combined to give a group answer (Assumption 14). There are too many topics for an individual student to learn exhaustively so that each student can only prepare in a way that allows them to achieve a fixed, individual accuracy acc*_k_*. How should students optimally prepare for this exam?

If students knew beforehand which groups they would be assigned to, they would do best to divide the topics and prepare only their assigned part. But such coordination is impossible (Assumption 13), here, because group assignment takes place after the preparation phase. Nevertheless, specialization is still the optimal strategy! Despite the possibility of overlapping specializations, the expected group accuracy is maximized by independent specialization because preparing a few topics exhaustively increases Type 2 performance. This allows students to better combine the individual responses during the exam and improves the group accuracy.

### Large Impact of Individual Type 2 Performance on Group Accuracies

**Figure 8.**
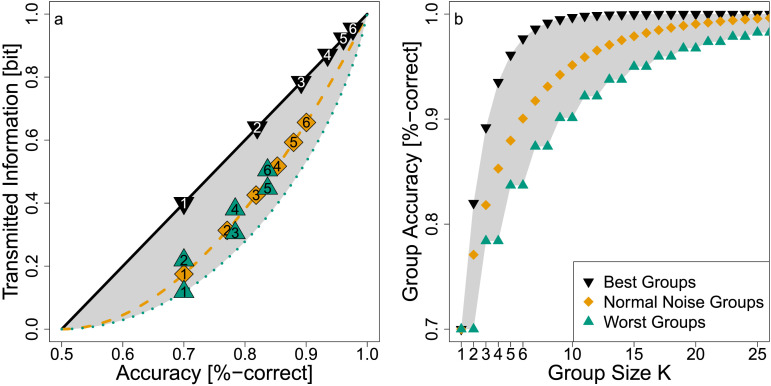
Individual Type 2 Performance and Group Accuracy. *Note*. Starting with individual classifiers with accuracy 70% but different underlying noise types (symbols with label 1), combining multiple such classifiers increases the accuracy. For example, optimally combining three classifiers with individual accuracy 70% and each with underlying uniform noise produces the group classifier indicated by the black inverted triangle 3. Similarly, for three normal noise classifiers (orange diamond 3) and three binary noise classifiers (green triangle 3). The rate at which group accuracies increase depends on the underlying noise type and with that on the transmitted information (*y*-axis) of the initial individual classifiers. In (b) we show the group accuracy trajectories with respect to the group size.

The difference between best and worst case group accuracy can be substantial. Figure 8 demonstrates this and relates the group accuracy to the information bounds established in Figure 3: Combining *K* = 3 classifiers with individual accuracies of acc_1_ = acc_2_ = acc_3_ = 70% with the same noise, yields different group accuracies depending on the noise case. A group of *K* = 3 classifiers with underlying uniform noise (RMI = 1) yields the highest group accuracy of acc_PCWMV_ = 89.2% (black inverted triangle 3) while a group of *K* = 3 classifiers with underlying binary noise (RMI = 1) yields the lowest group accuracy acc_PCWMV_ = 78.4% (green triangle 3). In contrast, normal noise classifiers yield an intermediate accuracy of acc_PCWMV_ = 82% (orange diamond 3).

Interestingly, groups of uniform noise classifiers and groups of normal noise classifiers can be represented by an individual classifiers with uniform and normal noise, respectively (with the corresponding increased accuracy). In contrast, binary noise classifiers diverge from the lower bound because, in a group, they allow for uncertainty differentiation: When most predict the same label there is high certainty and when predictions are close to a tie there is low certainty. Note that the tie-breaker problem leads to the step-like pattern of green triangles in Figure 8b because adding an even-numbered classifier will not improve accuracy but only increase Type 2 performance allowing the next odd classifier to actually increase the accuracy. Note also that binary noise classifiers approach the normal-noise trajectory (dashed, orange line in Figure 8a)—which is to be expected from the central limit theorem (e.g., see James, 1998)—but they climb at a much slower rate than groups of normal noise classifiers.

These results show that individual Type 2 performance has a substantial effect on the Type 1 performance of groups: Individual classifiers that maximize (vs. minimize) their Type 2 performance—measured by our RMI—also contribute most (vs. least) to the group’s Type 1 performance, even if their accuracy is held constant.

## DISCUSSION

We have taken a general perspective on classifiers, be it human raters or machine learning algorithms, that make predictions about a true label and give predictionwise confidence ratings. We have made the accuracy-information ambiguity explicit: For a fixed accuracy with which a classifier correctly predicts the true label, its transmitted information about the true label can take values in a large range. This range corresponds to the Type 2 performance of a classifier and we have derived a new measure, the relative metainformation RMI. This measure is normalized within the upper (RMI = 1) and lower information bounds (RMI = 0) making a step towards decoupling Type 2 from Type 1 performance. Thus, RMI is always the percentage of maximal information a classifier’s confidence ratings could convey in the ideal case (conditional on the Type 1 performance). This interpretation holds regardless of the classifier’s accuracy. Nevertheless, a full evaluation of RMI requires taking the Type 1 performance (e.g., accuracy) into account to contextualize Type 2 performances among different classifiers. This is so because a change in RMI could reflect a change in the noise distribution with the same accuracy (e.g., from normal to uniform noise) or a change in accuracy for the same noise (e.g., underlying normal noise for different accuracies). Additionally, we have extended work on group performance to formalize that the individual Type 2 performance has a substantial impact on the Type 1 performance in groups.

Note that we have referred to the information-theoretic measures as measures of Type 2 performance rather than measures of *metacognition*. This is because these measures only evaluate the match between true labels and classifiers’ responses (predictions and confidence ratings). Whether this match relates to the classifiers’ ability of metacognition is a matter of validity. For human raters in experimental tasks requiring a metacognitive representation, it may be appropriate to refer to them as a measure of metacognition (Fleming & Lau, 2014; Maniscalco & Lau, 2014). In other tasks or with non-human raters such as algorithmic classifiers, confidence ratings may not reflect metacognitive states but rather only signal strength of the presented stimuli (Carruthers & Williams, 2022; Kepecs & Mainen, 2012; Metcalfe, 2003; Paulewicz et al., 2020). Nevertheless, evaluating how well confidence ratings can differentiate levels of uncertainty (Type 2 performance) is highly relevant for these classifiers as well (especially for groups).

### Information-Theoretic Measures Only Evaluate Uncertainty Differentiation

One aspect of information-theoretic measures of Type 2 performance requires careful interpretation. We had derived our bounds using the perfect calibration assumption (Assumptions 3 or 7) but, importantly, this assumption was not required for applying the information-theoretic measures. This is because confidence ratings are estimated based on the observed relative frequencies. Consequently, we could have written Theorems 3 and 6 and the associated proofs based on participants’ responses *R* (on which we make no assumptions) instead of calibrated confidence ratings *C*: To determine the bounds, we would have then set *C* = *P*(*Y*|*R*)—corresponding to the relative frequencies—and continued the proofs as they are. We chose to frame our theorems based on confidence ratings *C* instead to illustrate the tie to metacognition, for ease of understanding, and because the calibration assumption does become necessary for our group performance bound results. Thus, because we estimate relative frequencies, the practical application of our information bounds and the RMI measure does not require the assumption that participants’ confidence ratings are perfectly calibrated.

As a crucial consequence of this, information-theoretic measures of Type 2 performance ignore the meaning of the predictions of a classifier’s responses: A “high” confidence response could be evaluated to have less certainty than a “low” confidence response. Information-theoretic measures do not differentiate between this situation and one in which these response labels are swapped. Thus, these measures may be argued to miss the very essence of metacognitive ability altogether: the ability to give *appropriate* confidence labels. These measures do not evaluate the appropriateness of response labels but only if the classifier is able to give responses that are associated with different levels of uncertainty. Accounting for this situation, a cautionary step is to consider information-theoretic measures as a measure of the *potential for metacognition*—because the ability to differentiate levels of uncertainty is a prerequisite for giving appropriate and meaningful confidence labels. But note that this potential for metacognition may also depend on the experimental design and the variability of the presented stimuli (see Rahnev & Fleming, 2019, for a cautionary tale).

Future work on information-theoretic measures may modify this by including ordinal constraints with respect to the classifier’s response labels, which is done by the SDT-based measures. Another avenue is to evaluate the appropriateness of confidence labels separately and split metacognitive performance into two facets: (1) the ability to give responses associated with different levels of uncertainty and (2) the ability to label these responses appropriately. In the probability elicitation literature, these two facets are called (1) refinement vs. (2) calibration (DeGroot & Fienberg, 1983).

*Refinement* (DeGroot & Fienberg, 1982, 1983; Masnadi-Shirazi, 2013) or *resolution* (Bröcker, 2009; Schervish, 1989) conceptualizes what we here measured via the transmitted information in an ordinal way: One classifier is considered to be more refined than another when the response of the second can be expressed as a function *g* of the first. This function *g* effectively lumps together responses with different levels of uncertainty (merging confidence bins). This incurs a loss of information and at the same time reduces refinement. Using the data processing inequality, this relationship is easily formalized for two classifiers *A* and *B* with responses $\left({\hat{Y}}_{A},C_{A}\right)$ and $\left({\hat{Y}}_{B},C_{B}\right)$:

$$\underset{\text{If}\;B\;\text{is equally or more refined than}\;A}{\underbrace{\left({\hat{Y}}_{A},C_{A}\right)=g\left({\hat{Y}}_{B},C_{B}\right)}}\Rightarrow \underset{\text{then}\;B\;\text{transmits equally or more information than}\;A.}{\underbrace{I\left(Y;{\hat{Y}}_{A},C_{A}\right)\leq I\left(Y;{\hat{Y}}_{B},C_{B}\right)}}$$

Our Theorems 3 and 6 determined the most extreme classifiers for a fixed accuracy: There exists no classifier that is strictly more refined than one with underlying uniform noise and no strictly less refined classifier than one with underlying binary noise.

Relatedly, information-theoretic measures are sensitive to the particular choice of confidence bins. For example, a binning scheme as we used in our simulations ([50%, 75%) and [75%, 100%]) may produce different results than other binning schemes (such as [50%, 90%) and [90%, 100%]). Fewer bins and sub-optimal positioning can decrease information-theoretic measures of Type 2 performance. With that, information-theoretic measures do not effectively control for thresholding neither regarding the predictions nor the confidence ratings (i.e., Type 1 and 2 biases). Using continuous (visual analog) scales (Matejka et al., 2016; perhaps with a log-odds scaling, Phillips & Edwards, 1966) may alleviate this problem because the threshold placement is then not hidden within the classifier but can be done explicitly by the experimenter.

### Relation of Information-Theoretic Measures to Meta−*d*′ and *M*−ratio

Some of the established measures either build on the normal noise assumption (meta−*d*′ and *M*−ratio) or normalize with respect to the normal noise case (*M*−ratio and $\text{meta}-I_{1}^{r}$). But the normal noise case constitutes only an intermediate case, which is sandwiched between the uniform noise (best) case and the binary noise (worst) case (Figure 6). It is, therefore, not clear whether the normal noise case should be the point of reference. Moreover, when comparing the Type 2 performances of different types of classifiers (e.g., human raters vs. algorithmic classifiers) making the normal noise assumption may fit the former better than the latter and, therefore, bias comparisons. Measures based on fewer assumptions may be more appropriate. For this reason, our proposed measure RMI takes the boundary cases as reference points. This keeps the range of possible values of our measure constant across different accuracy levels and for any underlying model. It nevertheless remains necessary to contextualize RMI values with the corresponding accuracies. Moreover, it is important to keep in mind that RMI presupposes the whole theoretically possible range of transmitted information: In practice, classifiers (e.g., real human participants) may not be able to attain the maximal and minimal values and researchers may, therefore, prefer measures tailored to their use-cases.

Figure 6 reveals one aspect that should be accounted for when basing measures on the normal noise case: The normal noise case behaves similarly to the binary noise case for low accuracies (large overlap of orange diamonds and green triangles on the left) but it approaches the uniform noise case for higher accuracies (orange diamonds departing from green triangles and approaching black inverted triangles). Consistent with this observation, RMI values increase for the normal noise case with increasing Type 1 performance. Adequately interpreting SDT-based Type 2 performance measures requires a conceptual clarification for this observation.

Moreover, information-theoretic measures in our setting differ from meta−*d*′ and *M*−ratio in how they attribute information about the true labels that is contained in the responses. To understand this difference, consider for example a human rater that (almost) always gives the same prediction, but consistently responds with a high confidence rating if the prediction is correct and with a low confidence rating if the prediction is incorrect. The traditional methods would attribute a low Type 1 sensitivity *d*′ and a high Type 2 metasensitivity meta−*d*′, because confidence ratings well separate correct from incorrect predictions. However, this induces an undesirable trade-off between Type 1 and Type 2 performance: If the classifier inverted some predictions given with low confidence—which the classifier knows to be certainly incorrect—, Type 1 performance would increase and Type 2 performance would decrease. Such a trade-off was empirically observed by Desender et al. (2022). To mitigate this undesirable confound of Type 2 with Type 1 performance, Assumptions 4 and 8 in our setting enforce that all available information of the responses about the true labels is first attributed to Type 1 performance. In the example, this would lead to a high accuracy, which reflects the fact that the classifier did process the true labels to make its responses. The surplus information—stemming from the ability of the classifier to differentiate uncertainties in these optimally coded predictions—is then attributed to Type 2 performance. We discuss this aspect in more depth in Supplement E.

### Limiting Assumptions for Group Bounds

Note that our results are interesting not despite but *because* we made strong theoretical assumptions. Even if individual classifiers are perfectly calibrated (Assumption 3), their responses are conditionally independent (Assumption 13), and the group optimally combines individual responses (Assumption 14) there is a large range of possible values of the group accuracy. Under more realistic conditions, this range can widen even more. Our theoretical results provide a foundation for understanding the substantial role that individual Type 2 performance plays in groups.

Note that, while our Type 2 measure was independent of the calibration assumption, our group bounds critically depend on perfect calibration. There is a vast literature on calibration in machine learning and confidence calibration for algorithmic classifiers can be facilitated during the construction (Gal & Ghahramani, 2016; Gawlikowski et al., 2023; Lakshminarayanan et al., 2017) or repaired post hoc (Guo et al., 2017). But if individual classifiers in a group are imperfectly calibrated, the group performance will deteriorate because improper weight is given to their predictions (cf. Ben-Yashar, 2023). In the worst case, confidence ratings are so inappropriately biased that unweighted majority voting may outperform weighting approaches (Kuncheva & Rodríguez, 2014).

For human confidence ratings, Zhang and Maloney (2012) have shown that post hoc calibration can often be achieved by a linear mapping in log odds space: When transforming uncalibrated confidence ratings $\hat{C}$ to the estimated log odds $\hat{L}O=\log\left(\frac{\hat{C}}{1-\hat{C}}\right)$ then these are often linearly related to the true log odds $LO=\log\left(\frac{C}{1-C}\right)$, see also Zhang et al. (2020). Curiously, in CWMV and PCWMV, the optimal weighting of classifiers’ predictions are also log odds (Ben-Yashar & Nitzan, 1997; Ben-Yashar & Paroush, 2001; Berend & Kontorovich, 2014; Grofman et al., 1983). Moreover, when human raters give confidence ratings on visual analogue scales, they seem to be better calibrated when using log odds scales rather than linear probability scales (Phillips & Edwards, 1966). In combination, these three results hint towards an inherent log odds representation of confidence ratings in humans, possibly due to the advantages of their additivity (Dayan & Daw, 2008; Rao, 2004).

Note, however, that our group results are limited to the case of binary labels, *L* = 2. Only in this case, there is one unique classifier that is most refined for a given accuracy and one unique classifier that is least refined for a given accuracy. In the *L* > 2 case, there are different “routes” of refinement. One of these routes leads to the highest information-theoretic Type 2 performance (as given by our Theorem 6) but it is not clear to us whether this also constitutes the best group performance. Other work has tackled this question (Ben-Yashar & Paroush, 2001; Li & Yu, 2014), but we are not aware of results akin to the CWMV or PCWMV group accuracies we presented here.

### Implications of Group Bounds for Cue Combination Research

Given the generality of our setting, our bounds are applicable to a wide range of situations in which multiple sources of information are combined. Another application we have so far not mentioned is that of cue combination, a situation in which multiple sources of information are integrated within a single human rater (Gao et al., 2025; Meyniel & Dehaene, 2017; Schulz et al., 2023). The cue combination literature studies how humans make orientation judgement (Knill & Pouget, 2004; Landy et al., 2011; Trommershäuser et al., 2011), detect sounds (Schönfelder & Wichmann, 2013), estimate depth (Landy et al., 1995; Negen et al., 2018), and estimate size (Ernst & Banks, 2002) based on multiple cues (typically from different modalities). In many cases, humans are found to combine cues optimally when assuming underlying normal noise (corresponding to the trajectory along the orange dashed curve in Figure 8). But sometimes, humans deviate from this expectation (Rahnev & Denison, 2018). This can be due to sub-optimal combination of normal noise cues but, as our results demonstrate, it can also be due to optimal combination of non-normal noise cases. Without assuming normal noise (see e.g., Burr et al., 2009), our bounds provide a range in which optimal cue combination performance can be expected.

### Conclusion

We contributed to the search for measures of metacognition by incorporating tight bounds into a normalized measure which we call relative metainformation RMI. This measure constitutes a step towards the much needed decoupling of Type 1 and 2 performance in order to measure metacognitive ability independently of other aspects such as accuracy. We have further shown how our measure relates to the accuracy of groups, which substantially depends on whether the individual group members are able to differentiate uncertainties in their responses or not. Despite difficulties in interpretations remain, our theoretical bounds allow better evaluation of measures of metacognition and the performance of groups.

## Acknowledgments

We thank Dorothee Maria Barbara Sigg, Helen Alber, and Arthur Otte for their input during this project.

## Funding Information

This research was supported by the Deutsche Forschungsgemeinschaft (DFG/German Research Foundation) CRC 1233 “Robust Vision” project 276693517; the Institutional Strategy of University of Tübingen (DFG, ZUK 63); and the Cluster of Excellence “Machine Learning: New Perspectives for Science”, EXC 2064/1, number 390727645.

## Author Contributions

Sascha Meyen: Conceptualization (Lead); Formal analysis (Lead); Investigation; Methodology; Validation; Visualization; Writing – original draft; Writing – review & editing. Frieder Göppert: Conceptualization; Formal analysis; Validation. Carina Schrenk: Formal analysis; Validation; Visualization (Supporting); Writing – review & editing. Ulrike von Luxburg: Formal analysis; Funding acquisition; Project administration; Supervision; Writing – review & editing. Volker H. Franz: Conceptualization; Funding acquisition; Project administration; Supervision (Lead), Writing – review & editing.

## Data Availability Statement

All analysis codes to replicate the following results and to compute the discussed measures are available at https://osf.io/sydbp/. Computations were performed using R, version 4.3.2 (R Core Team, 2020).

## Supplementary Material


