# Supplementary material for: Information-Theoretic Measures of Metacognition: Bounds and Relation to Group Performance
Source: Open Mind (Camb). 2025 Oct 17;9:1728–62. doi: 10.1162/OPMI.a.40 (PMC12618015; doi:10.1162/OPMI.a.40)
Supplement: Supplementary file 1 [file opmi-09-1728-s001.zip › OPMI.a.40-Supplemental Figures/Figure_S01_Proof_idea.pdf]

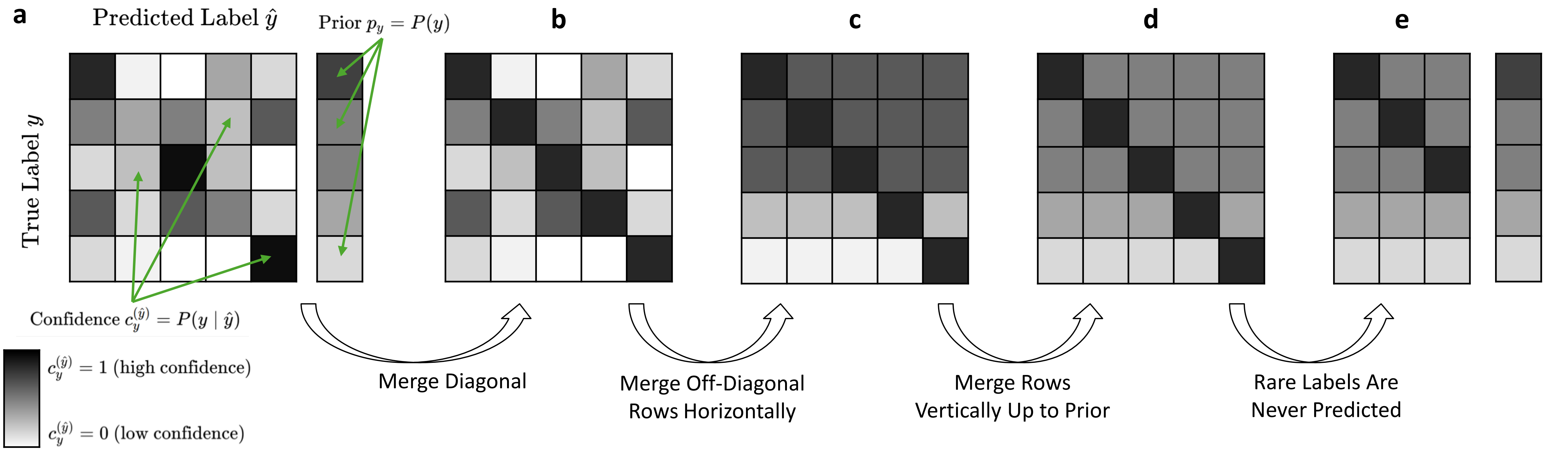
a
Predicted Label $\hat{y}$
Prior $p_y = P(y)$
True Label y
Confidence $c_y^{(\hat{y})} = P(y \mid \hat{y})$
$c_y^{(\hat{y})} = 1$ (high confidence)
$c_y^{(\hat{y})} = 0$ (low confidence)
b
c
d
e
Merge Diagonal
Merge Off-Diagonal Rows Horizontally
Merge Rows Vertically Up to Prior
Rare Labels Are Never Predicted
