# Supplementary figures and images for: Information-Theoretic Measures of Metacognition: Bounds and Relation to Group Performance
Source: Open Mind (Camb). 2025 Oct 17;9:1728–62. doi: 10.1162/OPMI.a.40 (PMC12618015; doi:10.1162/OPMI.a.40)

meta-d'

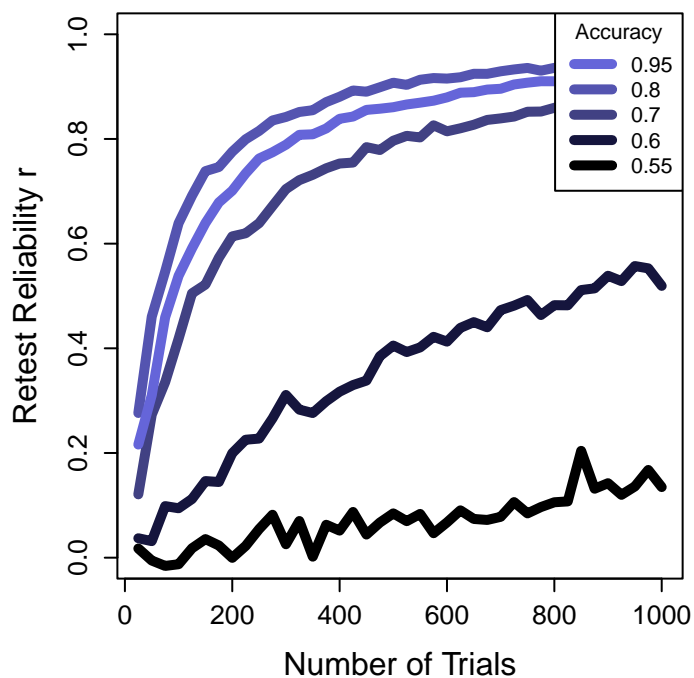

M-Ratio

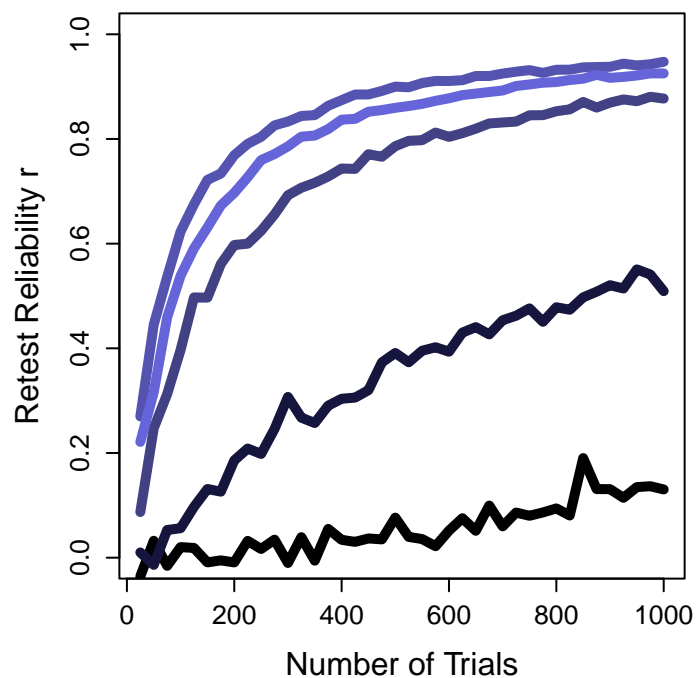

meta-I

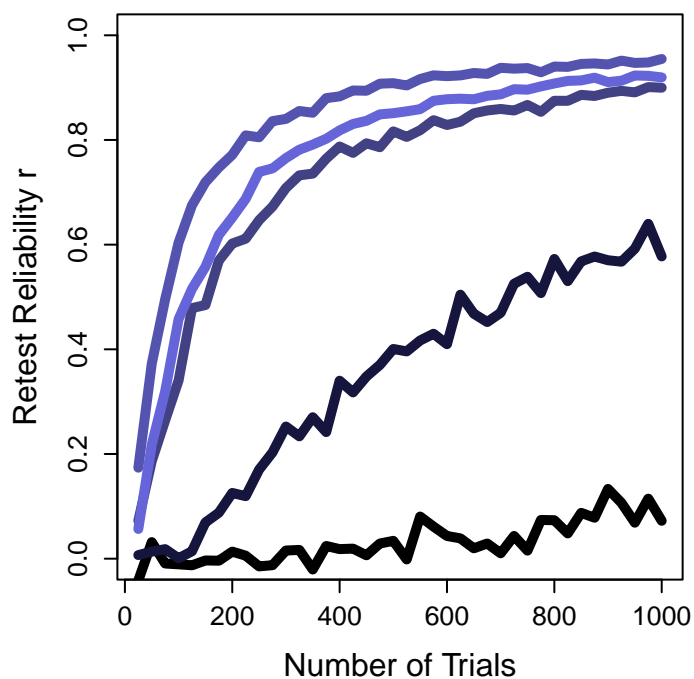meta-I<sub>1</sub><sup>r</sup>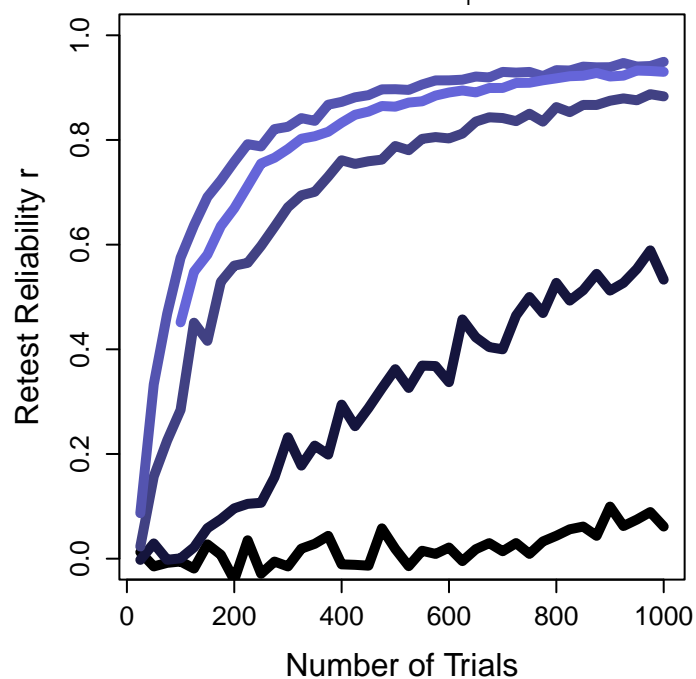meta-I<sub>2</sub><sup>r</sup>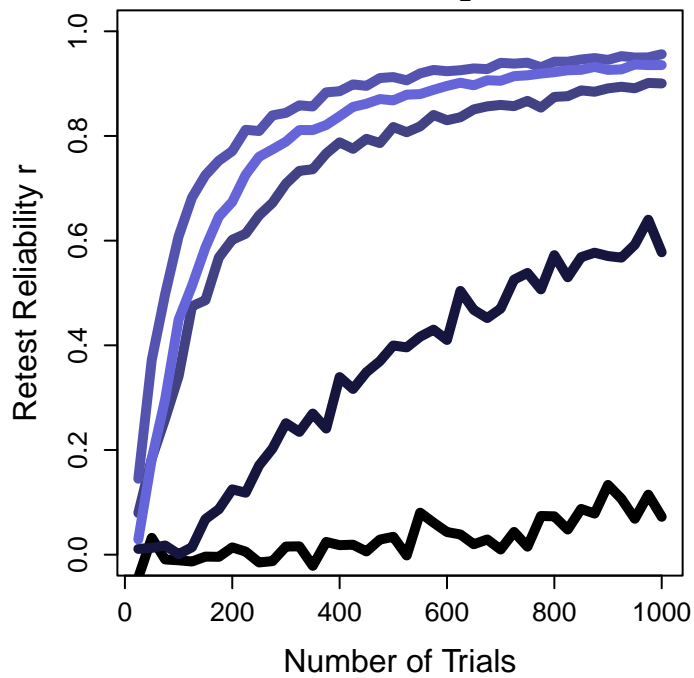

RMI

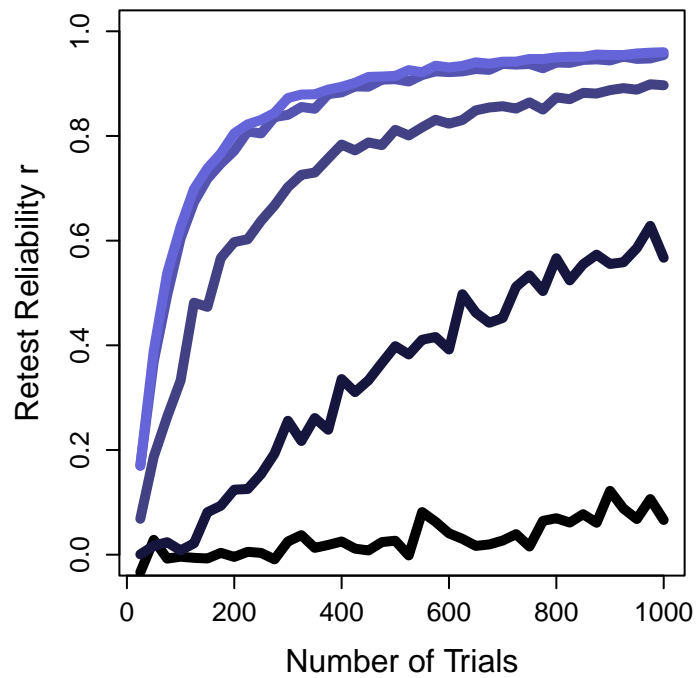

Supplement: Supplementary file 1 [file opmi-09-1728-s001.zip › OPMI.a.40-Supplemental Figures/Figure_S05_Reliability.pdf]

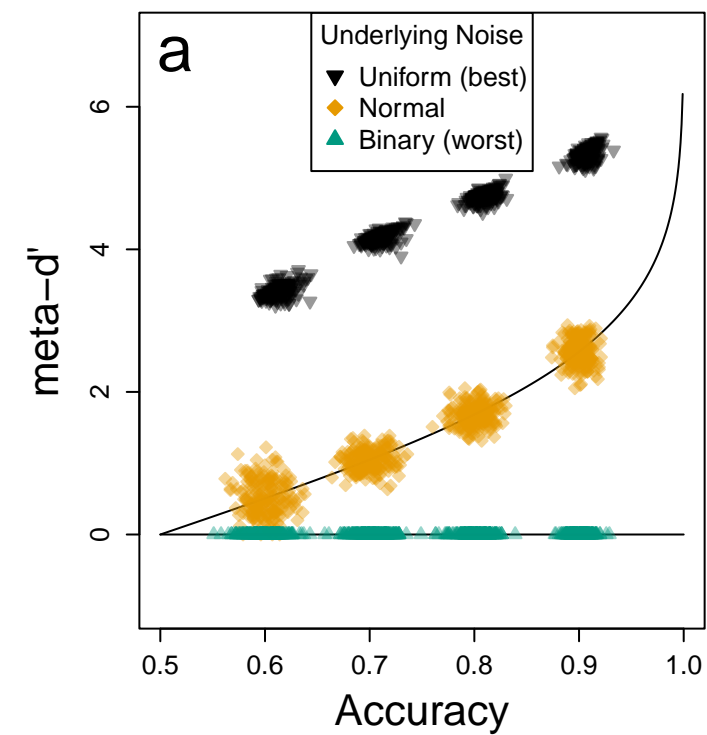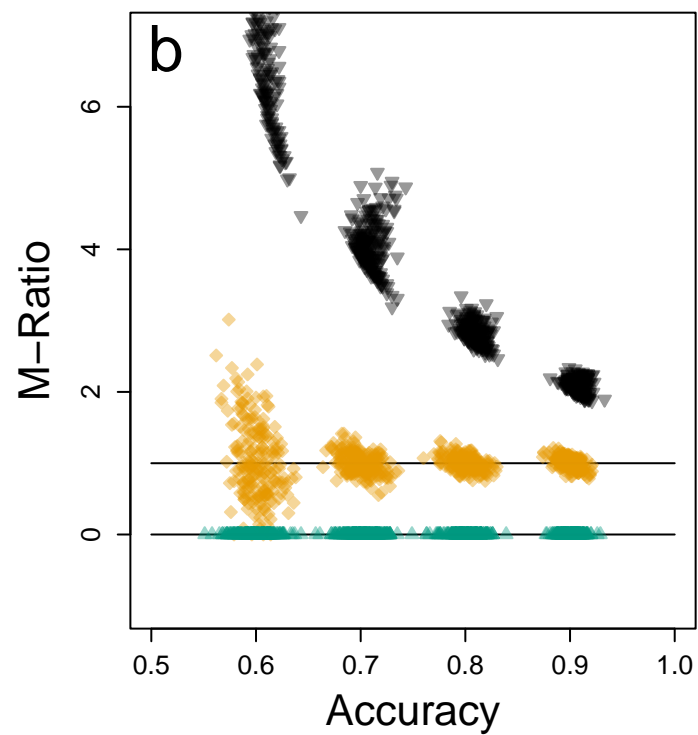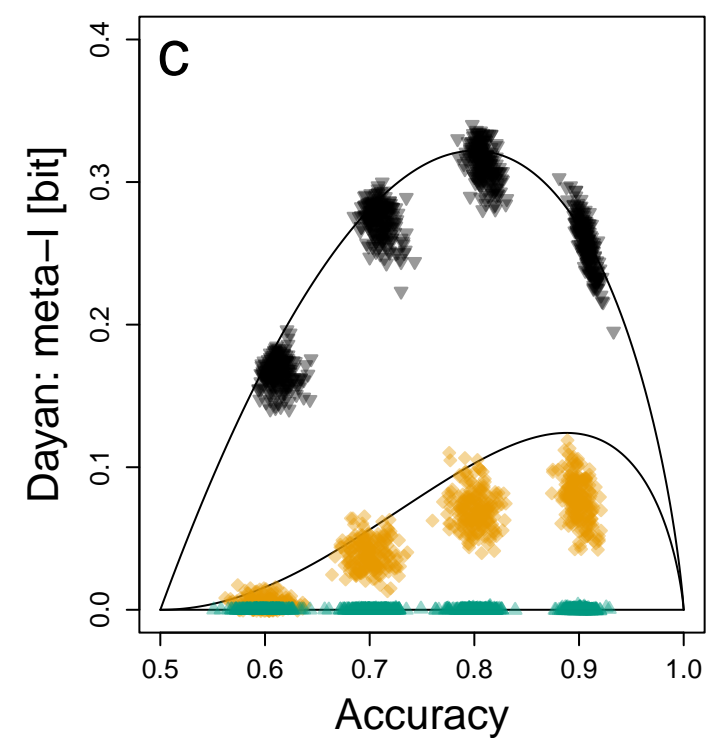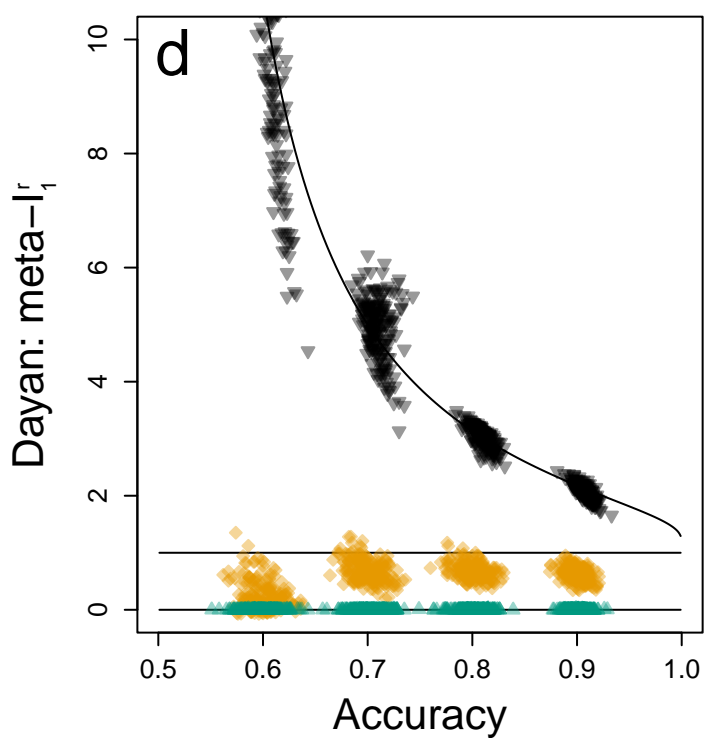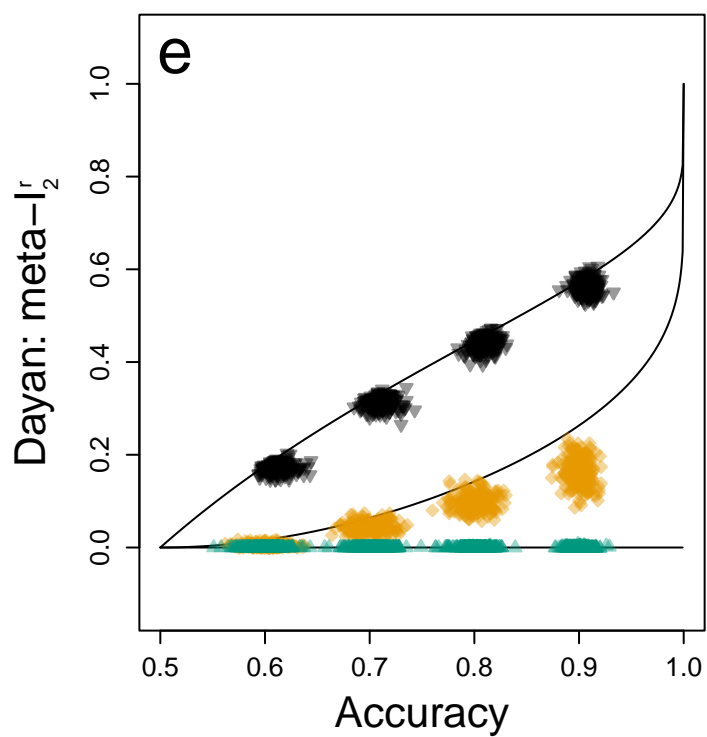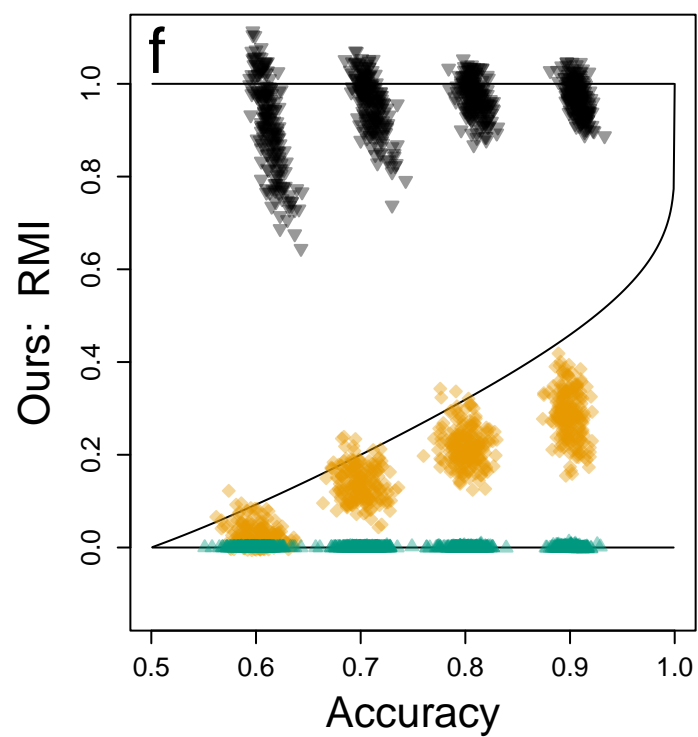

Supplement: Supplementary file 1 [file opmi-09-1728-s001.zip › OPMI.a.40-Supplemental Figures/Figure_S03_Metacognitive_measures_br_mo.pdf]

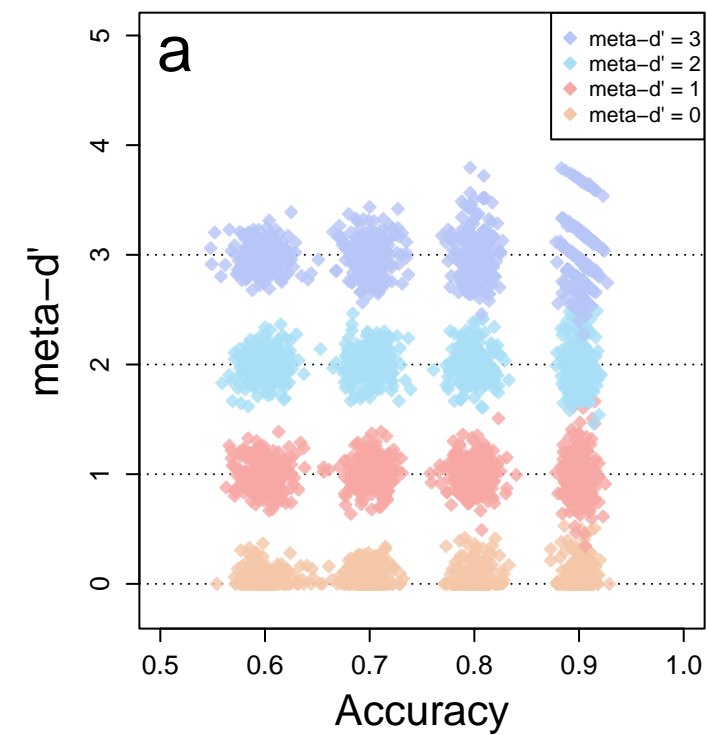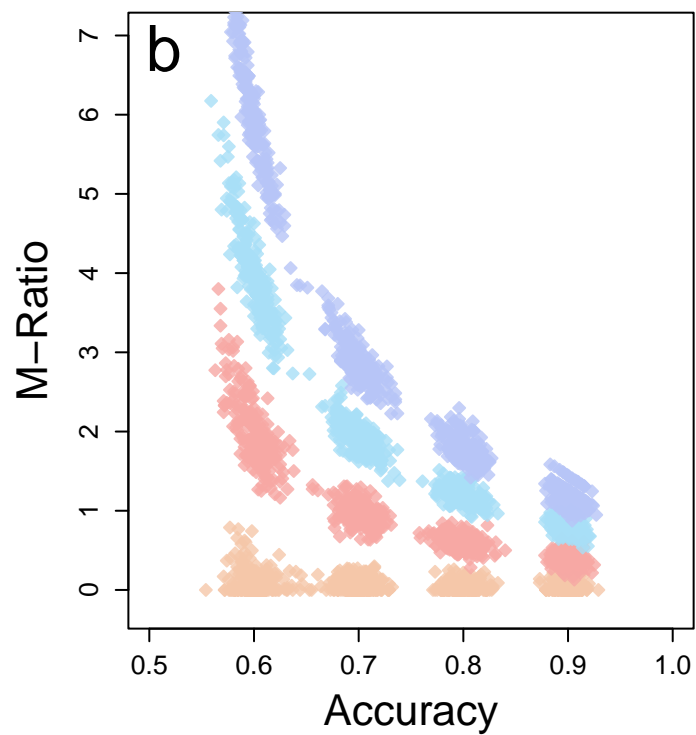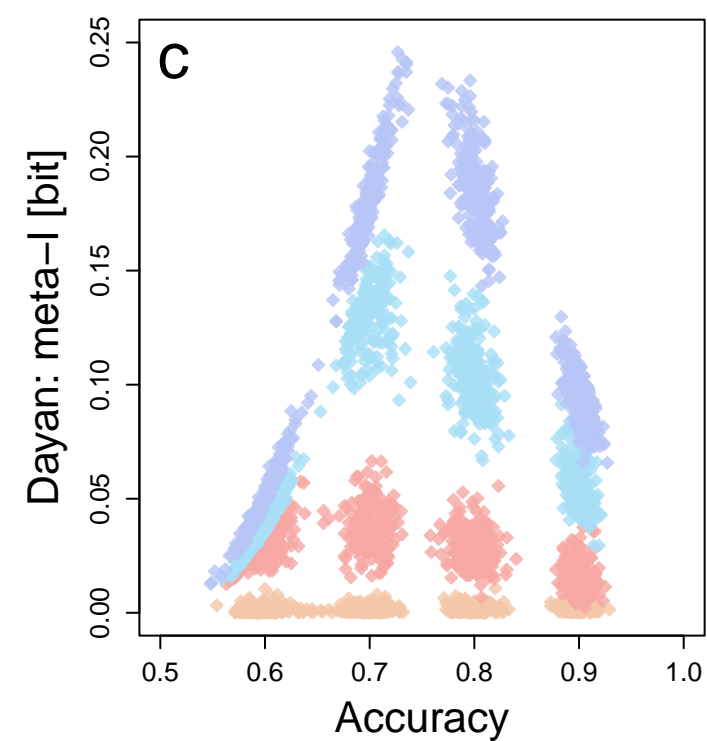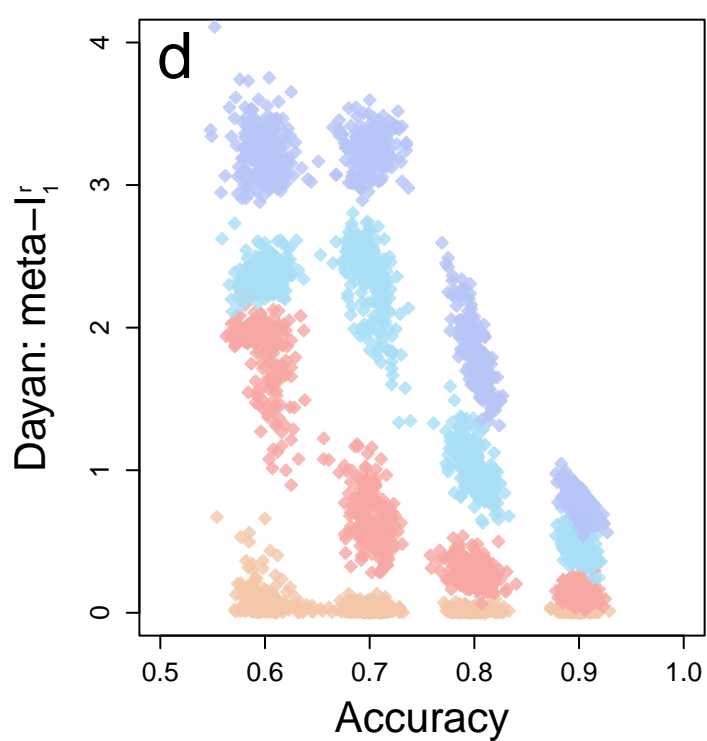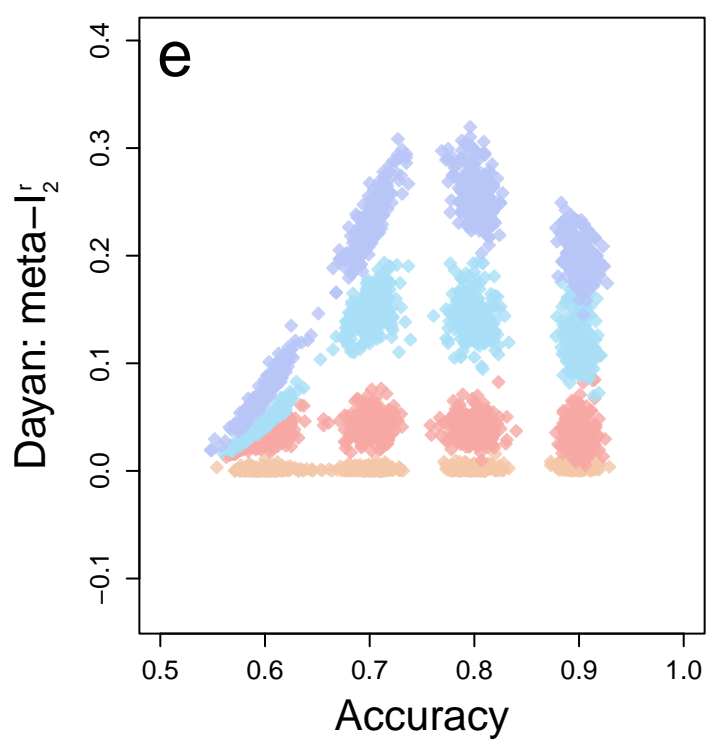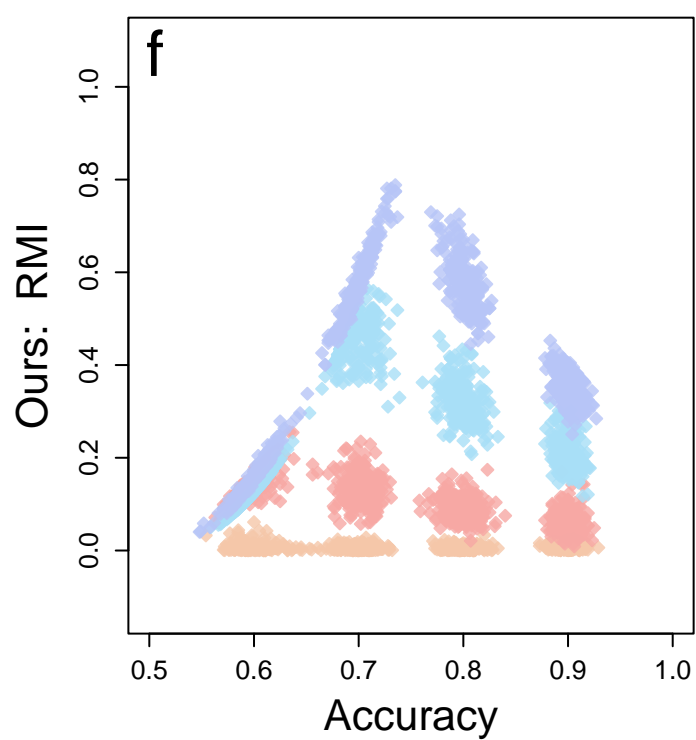

Supplement: Supplementary file 1 [file opmi-09-1728-s001.zip › OPMI.a.40-Supplemental Figures/Figure_S04_Truncated_normal_noise_simulations.pdf]

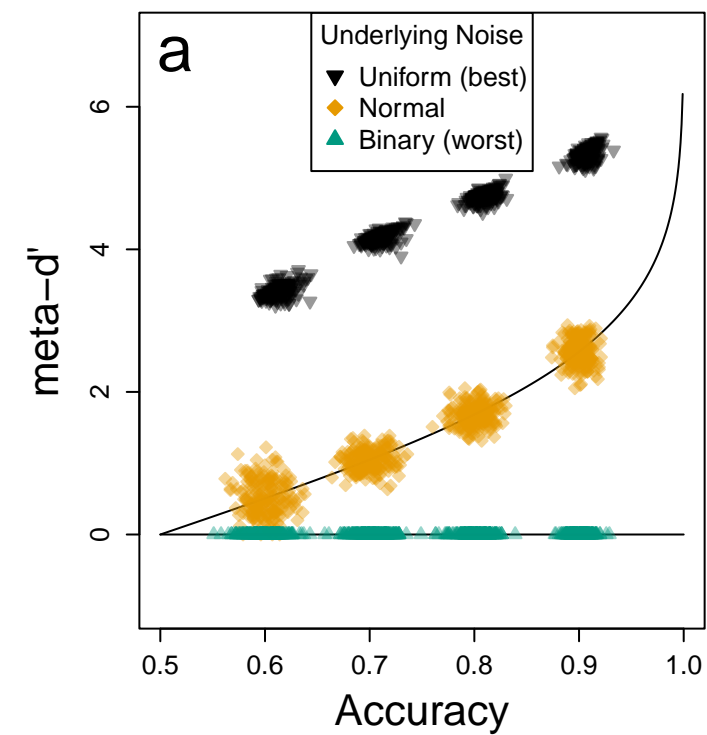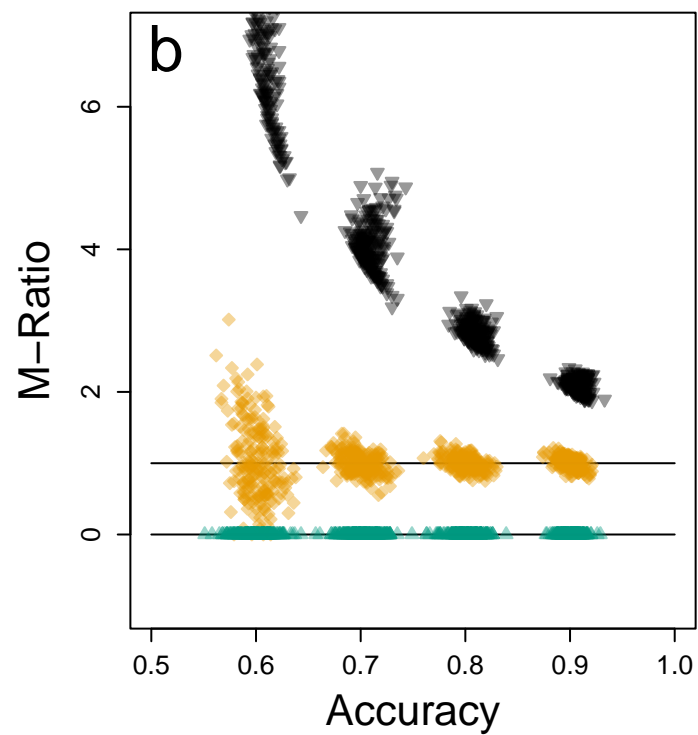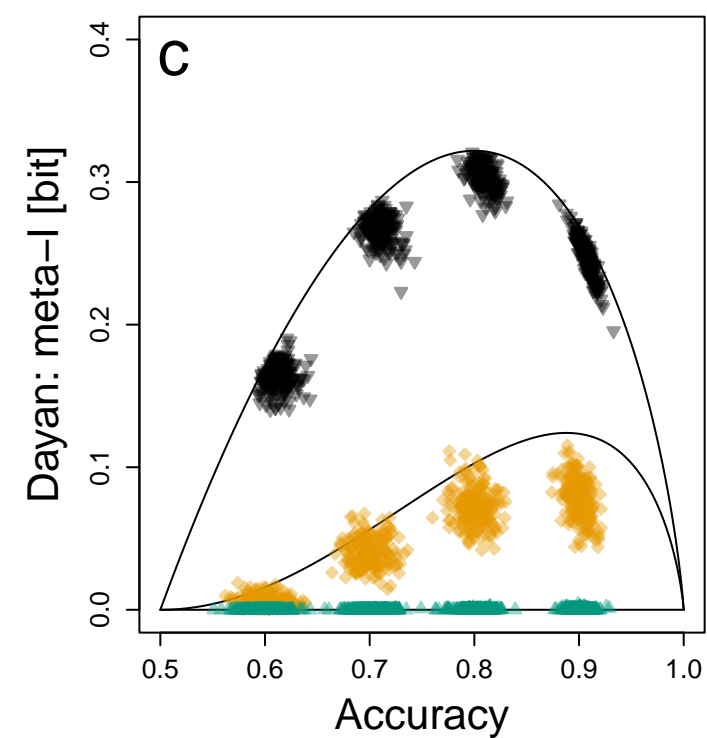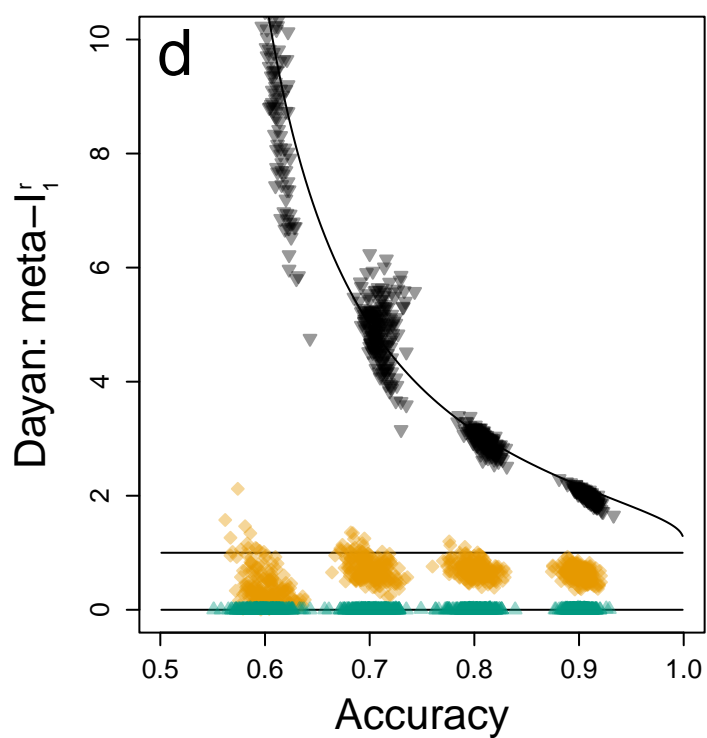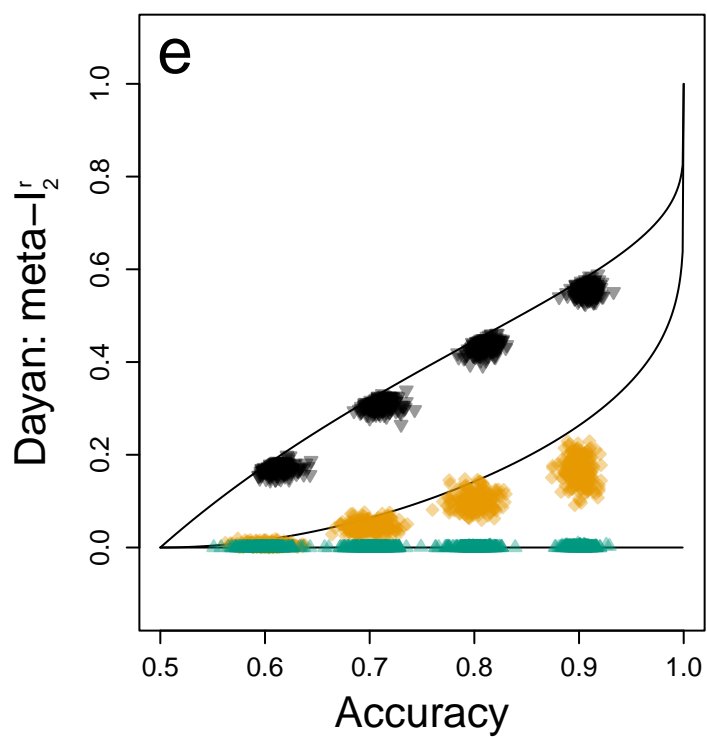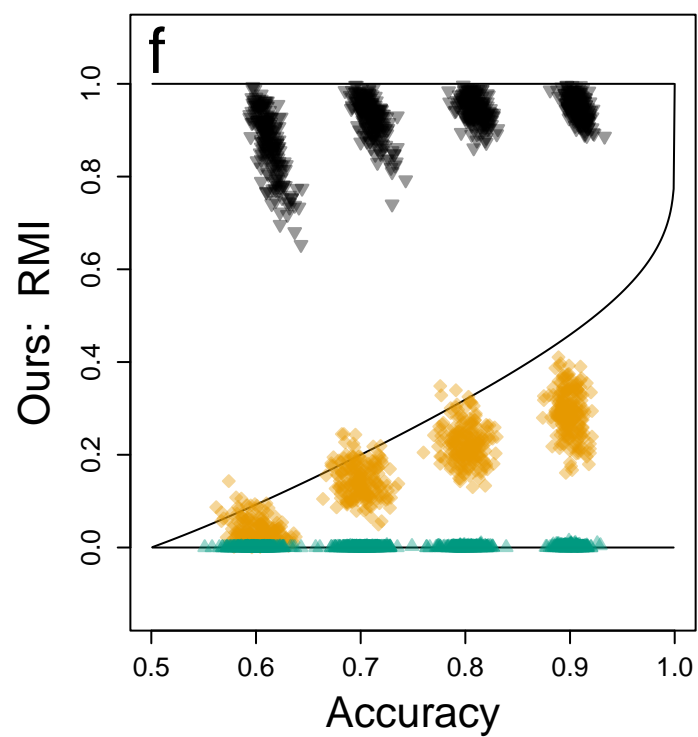

Supplement: Supplementary file 1 [file opmi-09-1728-s001.zip › OPMI.a.40-Supplemental Figures/Figure_S02_Metacognitive_measures_more_observations.pdf]
